# Psychosocial working conditions and violence prevention climate in German emergency departments – a cross-sectional study

**DOI:** 10.1186/s12873-024-01155-y

**Published:** 2025-01-23

**Authors:** Sonja Reißmann, Mannat Guliani, Tanja Wirth, David A. Groneberg, Volker Harth, Stefanie Mache

**Affiliations:** 1 Institute for Occupational and Maritime Medicine (ZfAM), University Medical Center Hamburg- Eppendorf (UKE), Seewartenstraße 10, 20459 Hamburg, Germany; 2 Institute of Occupational Medicine, Social Medicine and Environmental Medicine, Goethe University Frankfurt, Theodor-Stern-Kai 7, 60590 Frankfurt, Germany

**Keywords:** Emergency department, Working conditions, Workplace violence, Prevention, Violence prevention climate, Occupational safety

## Abstract

**Background:**

Emergency departments (EDs) are high pressure work environments with several psychosocial job demands, e.g., violence, and job resources, e.g., colleague support. So far, the perceptions of working conditions have been compared between doctors and nurses, but there is limited knowledge regarding their respective supervisors. In addition, the violence prevention climate has not been assessed in German EDs before. Thus, the current study focuses on differences in the perceptions of working conditions and the violence prevention climate between the groups of doctor-supervisors, doctor-employees, nurse-supervisors, and nurse-employees within the ED. Further analyses regarding the association between social relations and pressure for unsafe practices are performed, including the moderating role of belonging to one of the aforementioned groups.

**Methods:**

A cross-sectional online survey was carried out among *N* = 370 participants, who were doctors or nurses from German EDs. The Questionnaire for Psychosocial Risk Assessment (QPRA) and the Violence Prevention Climate Scale (VPCS) were applied. Kruskal-Wallis tests were performed for group comparisons, followed by a hierarchical multiple linear regression model and moderation analyses.

**Results:**

Statistically significant differences between the groups were found for eight out of 13 variables. The highest number of significant pairwise comparisons was found between the groups of doctor-supervisors and nurse-employees. High job demands regarding work intensity and work interruptions became apparent across all groups. Nurse-employees reported the highest social and emotional demands as well as the highest pressure for unsafe practices regarding violence prevention, significantly differing from the other groups on these variables. The variables of supervisor support and social stressors were found to be significantly predictive of pressure for unsafe practices. Furthermore, there was no moderating effect of belonging to one of the above-mentioned groups in the relationships between variables of social relations and pressure for unsafe practices.

**Conclusions:**

Differences found in the current study can help tailor preventive measures according to the needs of distinct professions and positions in order to improve working conditions and the violence prevention climate in EDs. Furthermore, supervisor support should be strengthened while social stressors should be resolved in order to decrease pressure for unsafe practices regarding violence prevention.

**Supplementary Information:**

The online version contains supplementary material available at 10.1186/s12873-024-01155-y.

## Background

Emergency departments (EDs) are high-pressure work environments [1]. A literature review on staff perceptions of the working environment in EDs has identified several key stressors, e.g., in terms of leadership and management (e.g., lack of support), communication (e.g., stressors regarding inter-professional communication), as well as workload and time pressure [2]. In addition, EDs have been described as emotionally challenging workplaces, with triggers on the level of patients (e.g., abusive behaviour), hospitals (e.g., limited resources), and the system (e.g., lack of community services) [3]. On the other hand, several job resources for ED staff have also been documented, such as social support and reward [4, 5]. Despite working in the same setting, several job demands and job resources related to psychosocial working conditions in EDs have been shown to differ between doctors and nurses [6–10]. However, there appears to be a research gap when it comes to comparisons that include the supervisors’ perspectives. Both, doctor [11] and nurse leaders [12] have additional tasks and also stressors related to management. Thus, their perceptions of working conditions might differ. Here, the first research question emerges: Are job demands and resources related to the psychosocial working conditions perceived differently by the groups categorised according to profession and position, i.e., doctor-supervisors, doctor-employees, nurse-supervisors, and nurse-employees in EDs?

The psychosocial job demand of being confronted with aggressive behaviour can be assessed as part of social and emotional stress [13]. Amongst healthcare professionals, this is specifically relevant for ED staff as they have been reported to be particularly affected by violence committed by patients and attendants [14]. In a recent study among doctors, nurses, and paramedics from German EDs, the 12-month prevalence for verbal violence perpetrated by patients and their attendants has been 97.1% and 94.3%, respectively. Within the year prior to the survey, participants have also experienced physical violence committed by patients (87.4%) and attendants (64.5%) [15]. According to the International Labour Organization (ILO), violence and harassment at work have been defined as “[…] a range of unacceptable behaviours and practices, or threats thereof, whether a single occurrence or repeated, that aim at, result in, or are likely to result in physical, psychological, sexual or economic harm […]” [16].

ED staff has been found to perceive violence as an inevitable part of their job while at the same time, they have reported a lack of preventive measures and support from direct supervisors and management [17]. Hence, supervisor support, e.g., concerning incidence reporting, has been demanded in the context of violence prevention [18], while co-worker support has been reported as a versatile measure of dealing with potentially aggressive patients [19]. Furthermore, leadership has been considered as a climate antecedent, proposing that leaders are the ones creating climate [20]. The violence prevention climate focuses on how employees perceive the organisational endeavours to fight workplace violence, i.e., placing policies and procedures against workplace violence and reducing factors contributing to pressure for unsafe practices (e.g., prioritising safety over productivity) [21]. To the authors’ best knowledge, the violence prevention climate has not been researched in German EDs so far [22], neither regarding differences in perceptions between groups according to profession and position, nor its relationship with factors of social support. Thus, three more exploratory research questions evolve, to expand the current body of research: Does the perception of the violence prevention climate differ between doctor-supervisors, doctor-employees, nurse-supervisors, and nurse-employees in EDs? And is there an association between job demands and job resources pertaining to social relations on the one hand (e.g., support of supervisors or colleagues) and pressure for unsafe practices regarding violence prevention in EDs on the other hand? Moreover, are these associations moderated by belonging to a certain group in terms of profession and position?

### Theoretical framework

The aim of this study is to explore perceptions regarding psychosocial job demands and job resources as well as the violence prevention climate among doctors, nurses, and their direct supervisors working in German EDs. Therefore, the study is based on two conceptual models: firstly, the Job Demands-Job Resources model (JD-R model) according to Bakker and Demerouti [23] and secondly, the violence prevention climate according to Kessler et al. [21].

#### Job demands-job resources model

The JD-R model is a flexible framework that can be applied in various work settings. It categorises working conditions into job demands and job resources, both of them being physical, psychological, social, or organisational aspects at work [23]. However, job demands require physical, cognitive, or emotional efforts and thus, are linked with physical or psychological costs, while job resources help to reach goals at work, facilitate personal growth, and are able to buffer high job demands [23]. According to the JD-R model, organisational outcomes are a result of two underlying psychological processes and their interactions: firstly, the health impairment process, where constant exposure to job demands is depleting resources and causing strain, or secondly, the motivational process, where availability of job resources is increasing motivation and work engagement [23]. The health impairment process and the motivational process have been described as mechanisms through which job demands and job resources are associated with safety outcomes, e.g., accidents, injuries, or unsafe behaviour [24].

#### Violence prevention climate

The violence prevention climate has been developed from the safety climate [25]. This concept is about employees‘ perceptions of organisational efforts against workplace violence [25]. A good perception of the climate can be assessed when organisations establish policies, which are also then diligently practiced to prevent and manage violence [21]. It further considers the role of supervisors in modelling interpersonal interactions and employees’ abilities to identify potential risk factors for violence [25]. While the current study focuses on violence committed by people outside of the organisation (i.e., patients and their attendants) the original concept is targeted at reducing aggression induced by both employees and others [25]. The multidimensional construct of the violence climate has been established by Kessler et al. [21], and is now called violence prevention climate [26]. The first two dimensions are policies and practices, which taken together create a positive violence prevention climate. The dimension of policies covers employees’ ratings on the availability of preventive measures, as well as training and information about them [21]. The dimension of practices captures employees‘ perceptions of how the management sticks to these policies and how it responds to reports of incidents of violence, as instating policies alone might not be sufficient if the management doesn’t adhere to them [21]. The third dimension of pressure for unsafe practices encompasses the perception of pressure felt by the employees to ignore violence prevention policies or procedures to satisfy their job requirements [21].

### Current state of research

The current study is based on previous findings concerning psychosocial working conditions and the violence prevention climate, which are presented in the following.

#### Psychosocial working conditions

##### Social relations

A network analysis from an Australian ED has shown that, despite ED staff being conceptualised as one large team, connections within problem-solving and socialising networks are closer among colleagues from the same profession [6]. In addition, more ties have been found between nurses compared to doctors in both networks [6].

Even though researchers have concluded that feedback and social support are important to facilitate employee well-being and forestall burnout in ED staff [5], several circumstances impede the provision of feedback in EDs, e.g., work pressure, communication failure, or shift changeover [27]. In EDs, a culture of people being quick in blaming others rather than supporting them has been described, e.g., when making a mistake or not being able to deal with high pressure, especially top-down from senior to junior colleagues [28]. Peer support has been found to be an important resource – although with limited opportunities to be exercised, whereas clinical leaders and management have been described as overburdened and not receptive to voiced concerns [28].

Medical [11] and nursing [12] leaders have a variety of tasks and juggle both healthcare and management. In addition, they work in a highly structured and hierarchical environment [29], being alone in their position without mentoring [28]. Although research on ED leadership is still in its early stages [30], a recent qualitative study has highlighted the important role of leaders in the ED regarding work culture, environment, practices, and behaviours [28]. Thus, the authors have concluded that unsupported and compromised leadership is a missed opportunity for a positive change of culture needed to improve working conditions and retain staff in EDs [28].

In summary, leaders’ support systems might be different from that of employees. With the objective of comparing job demands and resources pertaining to social relations among doctors, nurses, and respective supervisors in German EDs, the following assumption is proposed:

###### A1

With regard to social relations, the ratings for three job resources (A1a social support from colleagues, A1b social support from supervisors, A1c feedback and recognition) and for one job demand (A1d social stressors) differ significantly between the groups of doctor-supervisors, doctor-employees, nurse-supervisors, and nurse-employees.

##### Emotional load

Experiences of aggressive behaviour are a part of psychosocial job demands in terms of social and emotional stress [13]. Although the current study does not determine the prevalence of violence, it does assess participants’ perceptions of social and emotional demands caused by violent incidents, i.e., being confronted with aggressive or outrageous behaviour as well as emotionally draining situations (e.g., rage). The existing literature is heterogeneous when comparing the prevalence of violence between doctors and nurses. While there is literature stating that a larger proportion of nurses has experienced violence as compared to doctors [31–33], the other way around is also reported [34], sometimes depending on the type of violence [35, 36]. So far, there is more literature comparing different professions with regard to violence [14, 33, 35, 37], while there appears to be less literature comparing the different positions (supervisors and employees) [38, 39]. However, there is previous research on factors that might apply to persons in leadership positions. For instance, higher age has been found to be associated with less emotional violence in ED staff [31], and older healthcare workers in EDs have been found to have higher confidence in managing violence [40]. Similarly, less experienced ED staff has been more likely to be exposed to violence [38, 41].

Emotional labour has been described as an important part of working in EDs [42]. Several emotions of patients and attendants (e.g., fear, feeling overwhelmed or helpless) have been listed as causes of conflicts in EDs [43], highlighting the importance of emotional labour in the context of violence prevention. Rubin et al. have suggested emotional labour to be a response to perceived emotional dissonance, which in turn is the dissonance between the actual emotion felt and the emotion that is perceived as required [44]. Emotional labour has also been described in leadership positions of healthcare professions – and beyond that in two directions, i.e., with subordinate employees [45] and with superordinate leaders [46].

With the objective of expanding the knowledge on job demands related to emotional load in German ED staff, the current study analyses differences between doctors, nurses, and the respective supervisors in this regard. Therefore, the following assumption is suggested:

###### A2

The assessment of psychosocial job demands corresponding to emotional load, i.e., social and emotional demands (A2a) as well as emotional dissonance (A2b) differs significantly between the groups of doctor-supervisors, doctor-employees, nurse-supervisors, and nurse-employees.

##### Work organisation

EDs are high-pressure work environments [1] with a variety of stressors [2, 47] leading to health-related consequences for both staff and patients [48]. Several aspects of work organisation are contributory, for instance, high workload and time pressure [1, 2], working overtime [49, 50], workflow interruptions [51], multitasking [49], or working in shifts with insufficient breaks [52]. High pressure in EDs has been described for both professional groups, doctors and nurses [1, 2]. Differences between doctors and nurses have been observed regarding work tasks and concomitant factors, e.g., significantly shorter activity durations for ED nurses compared to ED doctors [10]. Furthermore, demands related to work organisation might vary between supervisors and employees. For instance, senior doctors have reported higher workloads than their junior colleagues [53], and nursing leaders have been described to have high workloads, with their overtime hours being linked to the quantity of employees they managed [54]. Consequently, the following assumption was formulated with the objective of comparing job demands related to work organisation between doctors, nurses, and their respective supervisors in German EDs:

###### A3

The appraisal of psychosocial job demands pertaining to work organisation (A3a work time design, A3b overtime, A3c work intensity, A3d work interruptions) differs significantly between the groups of doctor-supervisors, doctor-employees, nurse-supervisors, and nurse-employees.

#### Violence prevention climate

So far, there is limited literature on the violence prevention climate. However, regarding healthcare workers’ perceptions of the related safety climate, a study from Germany has found that nurses perceived higher occupational risks than doctors, while at the same time, nurses gave a more positive assessment of the employers’ occupational safety measures [55].

In contrast to supervisors, ED staff has been reported to have less opportunities to participate in the establishment of policies and procedures regarding violence prevention, e.g., due to a lack of contact persons or respective platforms [56].

Based on these findings, and with the objective of comparing perceptions of the violence prevention climate between doctors, nurses, and respective supervisors in German EDs, it is assumed that:

##### A4

The dimensions of the violence prevention climate (A4a practices and responses, A4b policies and procedures, A4c pressure for unsafe practices) are rated significantly different by the groups of doctor-supervisors, doctor-employees, nurse-supervisors, and nurse-employees.

#### Social relations and pressure for unsafe practices

An integral part to the concept of the violence prevention climate is the role of supervisors and co-workers, especially when it comes to pressure for unsafe practices: for instance, pressure might increase if supervisors place productivity as a higher value than safety, or if employees want to comply with group norms and observe their co-workers being indifferent to policies concerning violence prevention [21]. Previous research further indicates an association between social relations and violence. In a study among European nurses, good interpersonal relationships, e.g., with colleagues, charge nurses, and doctors, have been found to be a significant negative predictor of violence committed by patients and their relatives [57].

Concerning the related concept of safety climate, previous research has suggested that promoting an organisations’ safety climate can improve employees’ safety behaviour [58]. Job demands and resources (e.g., supervisor support) have been shown to be associated with healthcare workers’ safety behaviour, and the findings have further suggested that safety climate buffers the negative impact of job demands, and promotes the positive impact of job resources on safety behaviour [58]. In addition, social support has been shown to be positively related to the safety compliance of ED staff [59].

**Fig. 1:**
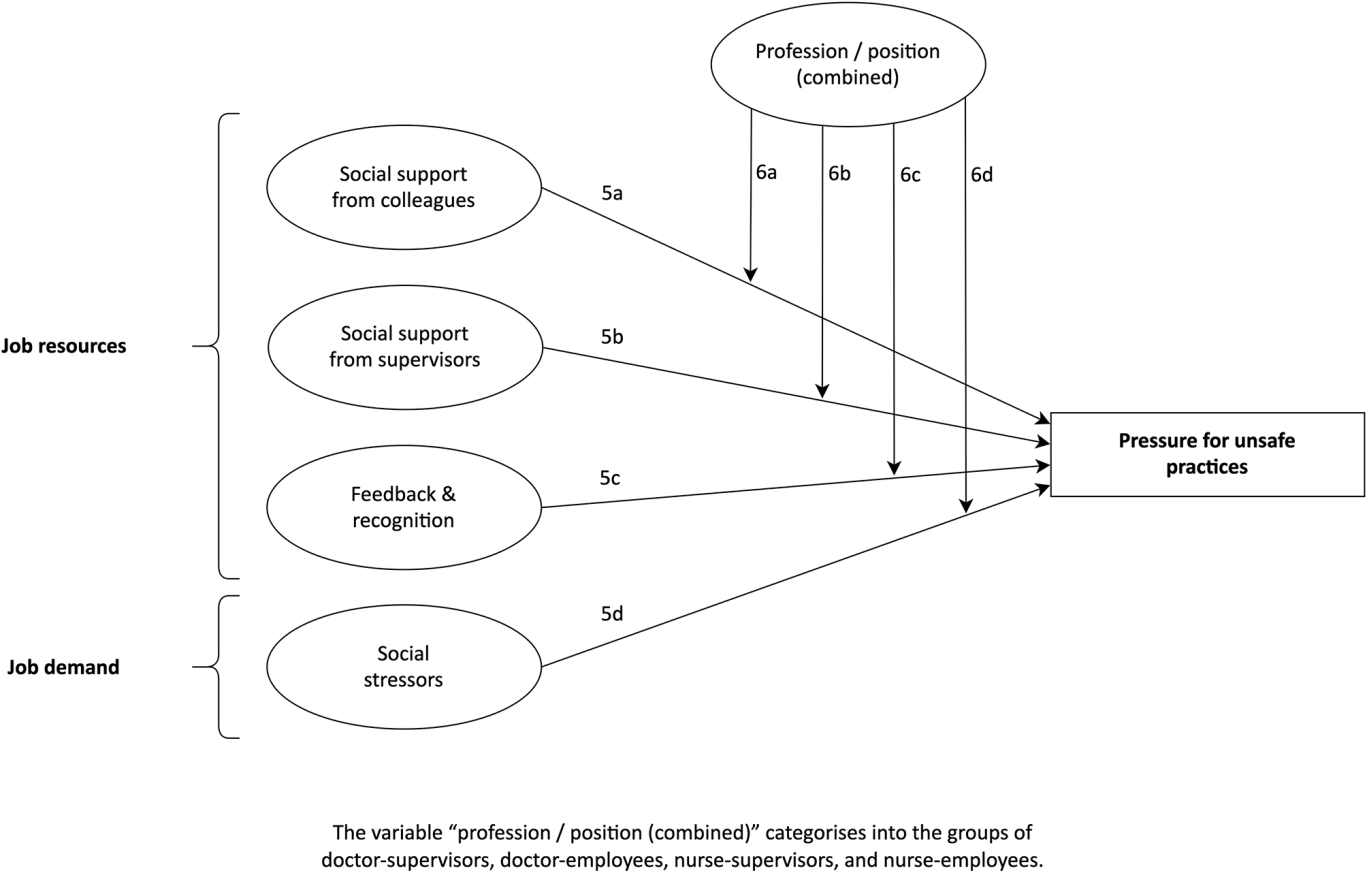
Conceptual model of assumptions regarding associations of social relations with pressure for unsafe practices (5a-d), as well as the moderating role of profession combined with position (6a-d)

Based on these findings and similar to the buffering effect of job resources according to the JD-R model [23], it is assumed that support of supervisors and colleagues, also in the form of feedback and recognition, can decrease the pressure to ignore violence prevention policies. Additionally, according to the health impairment process of the JD-R model [23], social stressors like conflicts between co-workers might increase the pressure for unsafe practices. Moreover, the research presented in previous sections is indicative of potential differences between the four groups concerning social relations. Consequently, the following exploratory assumptions are proposed with the objective of researching the associations between different aspects of social relations with pressure for unsafe practices in German EDs, as well as a potential moderating role of profession combined with position in these relationships (Fig. 1):

##### A5

The job resources as well as the job demand pertaining to social relations (A5a social support from colleagues, A5b social support from supervisors, A5c feedback and recognition, A5d social stressors) are significantly related to pressure for unsafe practices regarding the violence prevention climate, while controlling for membership in groups according to profession and position.

##### A6

Belonging to a specific group regarding profession and position (i.e., doctor-supervisors, doctor-employees, nurse-supervisors, and nurse-employees) moderates the associations of social relations (A6a social support from colleagues, A6b social support from supervisors, A6c feedback and recognition, A6d social stressors) with pressure for unsafe practices in terms of violence prevention.

## Methods

### Study design

A quantitative study was conducted, applying a cross-sectional design. The data were collected in Germany by means of an online questionnaire via the survey platform LamaPoll. Primary data were collected over a period of almost four months (17 November 2021 to 08 March 2022).

### Participants and recruitment

Four eligibility criteria were defined. Regarding the setting, the ED had to be an independent and spatially separated ward in the hospital. Participants had to have at least three months of work experience in the current ED, work in direct contact with patients, and practise either as doctor or nurse. These criteria were introduced to select participants with adequate experience in the current work environment and potential exposure to violence committed by patients or their attendants. Moreover, these criteria helped exclude employees who were exclusively performing administrative tasks or were mainly working outside of the ED in emergency rescue services.

Participants were recruited in two phases. In the first stage, a list provided by the Federal Joint Committee (German: Gemeinsamer Bundesausschuss, G-BA) was used, that assigns hospitals an emergency care level. The levels range from 0 to 3, with 0 meaning no participation in the emergency care level system or only specialised care modules (i.e., stroke), and with 3 denoting comprehensive care [60]. Taking the proportional quantity of hospitals per care level into consideration, 210 EDs (G-BA level 1 = 124, G-BA level 2 = 53, and G-BA level 3 = 33) were contacted. E-mails were sent to the respective medical and/or nursing leaders, asking to distribute the survey further among the ED staff. Level 0 hospitals were not contacted, as they may not have a spatially separated emergency ward. In the second phase of recruitment, e-mails were sent to 850 ED leaders or their representatives listed in the central directory of German EDs. This directory, aiming to list all EDs in Germany, was set up by the working group for Registry and Care Research from the Department of Traumatology (University Hospital Magdeburg) on behalf of the German Interdisciplinary Association for Intensive and Emergency Care (DIVI) and the German Society for Interdisciplinary Emergency and Acute Medicine (DGINA) [61].

A response rate could not be calculated since it was impossible to ascertain how many potential participants were reached by recruitment e-mails. During the period of data collection, the survey had 878 visitors, out of which 540 started the questionnaire, and 391 were registered as completed.

### Variables and measurement

Besides the measures listed below, the questionnaire applied in this study contained several scales from the health-oriented leadership instrument [62], the results of which are already published [22]. An overview of the complete questionnaire comprising a total of 104 items is provided in Table A (additional file 1).

All questions were mandatory to avoid missing data due to inadvertence. Hence, for questions to which participants might not know the answer (e.g., G-BA level) the option to select “not known” was provided.

#### Sociodemographic variables

Characteristics of participants and settings were assessed by means of self-constructed items. Regarding participants, information on age, gender, working hours, as well as work experience in the current ED and in EDs overall was collected. While profession was assessed as part of inclusion criteria (doctor or nurse, exclusion if selecting “neither”), leadership position was a binary yes/no question, providing the instruction for deputy leaders to only answer with “yes” if tasks and responsibilities were equally shared with nursing or medical ED leaders, respectively. Regarding the setting, G-BA level, number of hospital beds, type of hospital funding and location (federal state in Germany) were asked for.

#### Questionnaire for psychosocial risk assessment

The original German version of the Questionnaire for Psychosocial Risk Assessment (QPRA; German: Fragebogen zur Gefährdungsbeurteilung psychischer Belastungen, FGBU) was published by Dettmers and Krause [13]. It was developed with 19 subscales assessing psychosocial job demands and job resources complemented by an index of 10 psychologically relevant physical stressors. Each subscale comprises three items to be answered on a four-point Likert scale (1 = *not true*, 2 = *rather not true*, 3 = *rather true*, 4 = *true*). Higher values either indicate higher job demands or higher job resources, respectively. Out of the original 19 subscales, only ten were used in the current study. These were four subscales pertaining to social relations (social support from colleagues, social support from supervisors, feedback and recognition, as well as social stressors), two subscales pertaining to emotional load (social and emotional demands as well as emotional dissonance) and four subscales pertaining to work organisation (work time design, overtime, work intensity, and work interruptions). A sample item from the subscale of work intensity translated into English is: “The high volume of work often causes intense time pressure” [13, p. 119, in German]. All items were listed in the original German publication [13]. Dettmers and Krause published comprehensive results concerning validity and reliability testing based on three studies [13]. Internal consistency was reported in all three studies as Cronbach’s α ≥ 0.70 for the subscales relevant to the current research, with the exception of work time design, as its values were Cronbach’s α = 0.66 in one study, but Cronbach’s α = 0.85 in the other two studies [13]. Following Dettmers and Krause [13], mean values of the three items per subscale were calculated in the present study to obtain values ranging between 1 and 4.

#### Violence prevention climate scale

The original English version of the Violence Prevention Climate Scale (VPCS) was published by Kessler et al., comprising 18 items allocated to three scales with six items each, to be answered on a six-point Likert scale [21]. The scales were defined as practices and responses (e.g., “management encourages employees to report verbal violence”), policies and procedures (e.g., “my employer provides adequate assault/violence prevention procedures”), as well as pressure for unsafe practices (e.g., “in my unit, human resource shortage undermines violence prevention standards”) [21, p. 114]. The complete English scale can be accessed [26]. Kessler et al. assessed construct validity as well as criterion validity and calculated Cronbach’s α ≥ 0.90 for all three scales, demonstrating good internal consistency [21].

For this study, the English version was translated into German by native English and German speakers amongst the authors. After consultation with the VPCS’ copyright holder, the wording was slightly adapted to the context of this study, e.g., management was replaced with “hospital management” (German: Klinikleitung), and a five-point Likert scale was used (1 = *strongly disagree*, 2 = *disagree*, 3 = *neither agree nor disagree*, 4 = *agree*, and 5 = *strongly agree*). In compliance with the VPCS scoring instructions, sum values of the six items per scale were calculated to obtain scale values ranging between 6 and 30 [26]. According to the original authors, the items of pressure for unsafe practices should be reverse coded so that higher scores always represent a good climate [21]. Although, this approach was followed in the other publication from this study [22], it was chosen not to reverse the respective scale items in the present analysis to keep the results more easily interpretable (higher pressure equalling to higher scores).

### Statistical methods

In the first step, the data set was cleaned by checking for completeness and plausibility of answers.

Out of those listed as completed (*N* = 391), participants not satisfying the above-mentioned inclusion criteria (*n* = 14) and participants who straightlined across the scales of a complete instrument (*n* = 7) were removed from the dataset. The final sample comprised *N* = 370 participants.

The statistical analyses described below were carried out for the total sample or in groups considering profession and position as the classifying variables, resulting in the groups of doctor-supervisors, doctor-employees, nurse-supervisors, and nurse-employees.

Descriptive statistics for participant characteristics and hospital data were compiled. Measures of central tendency and dispersion were calculated for all metric variables, in the total sample and divided into groups. Furthermore, Cronbach’s α was computed for each (sub-)scale.

Subsequently, the data were checked for normal distribution and outliers in the total sample as well as divided into the above-mentioned groups. For all metric variables, normal distribution was checked considering histograms, Q-Q plots, skewness, as well as kurtosis, and outliers were detected using boxplots and z-scores. Answers of participants showing z-scores larger than ± 3 or extreme outliers in boxplots were inspected for plausibility. Eventually, no outlier was excluded as these were considered as valuable perspectives. The assumption of normal distribution was found to be violated. Hence, in the next step, non-parametric Spearman’s correlation coefficients were used to calculate correlations of all metric variables. For this analysis, bootstrapping based on 1,000 samples was performed and bias-corrected and accelerated confidence intervals (BCa CI) were obtained.

According to A1-A4 the four groups were compared across all 13 variables of QPRA and VPCS. As the assumption of normal distribution was violated, the non-parametric Kruskal-Wallis test with a Monte Carlo method based on 100,000 sampled tables was chosen. Beforehand, homogeneity of variance was verified using the Levene’s test, supplemented by the variance ratio. To adjust the resulting *p*-values for multiple comparisons, the Holm-Bonferroni procedure was used. Follow-up analysis was conducted using pairwise comparisons through the Dunn-Bonferroni test preconfigured in SPSS. In the following, unadjusted *p*-values were taken from the output and adjusted by applying the Holm-Bonferroni procedure to address family-wise type I error. Effect sizes were calculated for pairwise comparisons using Pearson’s correlation coefficient, with *r* = 0.10 indicating a small, *r* = 0.30 denoting a medium, and *r* = 0.50 suggesting a large effect according to Cohen [63].

In preparation of regression and moderation analysis, it was verified whether the underlying assumptions were met. Thus, scatterplots were visually inspected for linearity and homoscedasticity; correlation matrix, variance inflation factor (VIF), as well as tolerance statistic were assessed with regard to multicollinearity, and independence of residuals was determined by means of the Durbin-Watson statistic. Normally distributed residuals were inspected with histograms and P-P plots. Outliers were detected based on standardised residuals, but retained in the sample after the respective answers were checked for plausibility and as no influential cases were determined using Cook’s distance.

All assumptions were met, with one potential restriction regarding moderation analyses: on two subscales of the QPRA, the assumption of linearity seemed slightly violated for certain groups, as the locally estimated scatterplot smoothing (LOESS) curve seemed to deviate from linearity on both edges. These were the groups of doctor-supervisors and doctor-employees on the subscale of social support from colleagues, and doctor-employees on the subscale of feedback and recognition. However, these deviations may be explained by the small sample size of these groups and the large dispersion of datapoints. Furthermore, the other groups satisfied the assumption of linearity. Hence, all moderation analyses were conducted.

A5 was tested using a hierarchical multiple linear regression model with pressure for unsafe practices as the dependent variable and the four variables pertaining to social relations as independent variables. An a priori sample size was calculated to estimate the required number of participants, using G*power (version 3.1) for a multiple linear regression model with the following parameters: alpha value α = 0.050, power (1 – β) = 0.950, effect size *f*^2^ = 0.090, and seven predictors (including an indicator-coded control variable), amounting to a sample size of *N* = 250. As outlined in the current state of research, there is literature suggesting that the analysed concepts might be assessed differently by the groups according to profession and position. Thus, in order to control for profession and position, the multicategorical variable defining membership in one of the four groups was entered into the model as an indicator-coded variable. Following the guidelines provided by Becker et al. [64], the control variables were entered in the first block of hierarchical linear regression analysis. The important role of supervisors and colleagues regarding pressure for unsafe practices has been discussed by the authors of the VPCS [21]. Thus, the two predictors of support from supervisors and colleagues were entered simultaneously in the second block, based on theoretical rationale. In the third model, the third job resource (i.e., feedback and recognition) was entered, followed by the only job demand pertaining to social relations (i.e., social stressors) in the fourth and final model. Bootstrapping based on 1,000 samples was conducted to obtain BCa CI, as well as robust significance tests and standard errors. The effect size Cohen *f*^2^ was calculated for the final model, in order to assess whether the effect size was small (*f*^2^ = 0.02), medium (*f*^2^ = 0.15), or large (*f*^2^ = 0.35) [63].

According to A6 it was analysed if the multicategorical variable dividing the sample into four groups according to profession and position had a moderating role in the relationships between the respective subscales pertaining to social relations (predictors) and pressure for unsafe practices (outcome). For an estimate of the sample size required to test if including the interaction would significantly explain more variance, an a priori sample size was calculated using G*power (version 3.1) for a multiple linear regression model (*R*^*2*^ increase) with the parameters: alpha value α = 0.050, power (1 – β) = 0.950, effect size *f*^*2*^ = 0.090, one tested predictor and seven predictors in total (including an indicator-coded variable), amounting to a sample size of *N* = 147. Four separate moderation analyses were conducted. The moderator was indicator-coded, the predictors were mean-centred, the heteroscedasticity-consistent standard error estimator HC4 was applied, and bootstrapped confidence intervals based on 5,000 samples were obtained.

Data analysis was conducted using IBM SPSS Statistics (version 26 and 29) supplemented by PROCESS macro (version 4.2) for moderation analysis. Significance was assessed for non-directional assumptions, and *p*-values were considered significant if *p* ≤ 0.050. The STROBE statement (Strengthening the Reporting of Observational Studies in Epidemiology) was applied to ensure this research complies with the reporting guidelines for cross-sectional studies [65] (additional file 2).

## Results

### Descriptive statistics

The final sample consisted of *N* = 370 participants from German EDs, divided into the four groups of doctor-supervisors (*n* = 75; 20.27%), doctor-employees (*n* = 37; 10%), nurse-supervisors (*n* = 91; 24.59%), and nurse-employees (*n* = 167; 45.14%). Participant characteristics are displayed in Table 1. Specifications about the hospital setting can be found in Table B (additional file 1), for instance, 57% of respondents stated to work in publicly sponsored hospitals.

**Table 1:** Participant characteristics (*N* = 370)

Variables		***n***	%
Gender	Female	220	59.5
	Male	150	40.5
	Diverse	0	0.0
Age in years	20–29	84	22.7
	30–39	99	26.8
	40–49	80	21.6
	50–59	92	24.9
	≥ 60	15	4.1
Profession	Nurse	258	69.7
	Doctor	112	30.3
Position	Supervisor	166	44.9
	Employee	204	55.1
Working hours	Full-time (≥ 35 h / week)	298	80.5
	Part-time (15–34 h / week)	64	17.3
	Part-time (< 15 h / week)	8	2.2
Work experience in the current ED^a^	< 1 year	24	6.7
	1–5 years	147	41.3
	6–10 years	93	26.1
	11–15 years	39	11.0
	> 15 years	53	14.9
Total work experience in any ED^a^	< 1 year	14	3.9
	1–5 years	116	32.6
	6–10 years	91	25.6
	11–15 years	57	16.0
	> 15 years	78	21.9

^a^*N* = 356, as *n* = 14 participants were excluded for this question as they answered to have spent more time in their current ED in comparison to their total work experience in EDs. Thus, their answers were considered as not plausible

**Table 2:** Descriptive statistics of study variables for the total sample and in groups according to profession and position

	Variable in the total sample / variable per group^a^	M	SD	Mdn	IQR	Minimum^b^	Maximum^b^	Floor-effect^c^ (%)	Ceiling-effect^c^ (%)
**Questionnaire for Psychosocial Risk Assessment (QPRA)** ^**d**^									
***Social relations***									
1	**Social support from colleagues**	**3.14**	**0.64**	**3.00**	**0.67**	**1.00**	**4.00**	**1.08**	**20.54**
	Doctor-Supervisor	3.00	0.68	3.00	0.67	1.00	4.00	1.33	16.00
	Doctor-Employee	3.14	0.59	3.00	0.67	1.67	4.00	0.00	13.51
	Nurse-Supervisor	3.27	0.52	3.33	0.67	2.00	4.00	0.00	23.08
	Nurse-Employee	3.14	0.68	3.00	0.67	1.00	4.00	1.80	22.75
2	**Social support from supervisors**	**2.91**	**0.82**	**3.00**	**1.33**	**1.00**	**4.00**	**3.24**	**15.95**
	Doctor-Supervisor	2.86	0.78	3.00	1.00	1.00	4.00	1.33	16.00
	Doctor-Employee	3.20	0.69	3.00	0.83	1.00	4.00	2.70	21.62
	Nurse-Supervisor	2.99	0.77	3.00	1.00	1.00	4.00	3.30	15.38
	Nurse-Employee	2.81	0.87	3.00	1.67	1.00	4.00	4.19	14.97
3	**Feedback and recognition**	**2.48**	**0.78**	**2.33**	**1.00**	**1.00**	**4.00**	**4.32**	**6.22**
	Doctor-Supervisor	2.53	0.63	2.67	1.00	1.00	4.00	2.67	5.33
	Doctor-Employee	2.89	0.66	3.00	1.17	1.67	4.00	0.00	5.41
	Nurse-Supervisor	2.55	0.78	2.67	1.00	1.00	4.00	6.59	5.49
	Nurse-Employee	2.34	0.82	2.33	1.33	1.00	4.00	4.79	7.19
4	**Social stressors**	**2.04**	**0.66**	**2.00**	**0.67**	**1.00**	**4.00**	**6.49**	**1.89**
	Doctor-Supervisor	1.96	0.63	2.00	1.00	1.00	3.67	6.67	0.00
	Doctor-Employee	1.86	0.52	2.00	1.00	1.00	3.00	8.11	0.00
	Nurse-Supervisor	2.00	0.69	2.00	1.00	1.00	4.00	7.69	3.30
	Nurse-Employee	2.13	0.67	2.00	0.67	1.00	4.00	5.39	2.40
***Emotional load***									
5	**Social and emotional demands**	**3.21**	**0.62**	**3.33**	**1.00**	**1.33**	**4.00**	**0.00**	**18.92**
	Doctor-Supervisor	2.82	0.67	2.67	1.00	1.33	4.00	0.00	12.00
	Doctor-Employee	3.09	0.56	3.00	0.83	2.00	4.00	0.00	13.51
	Nurse-Supervisor	3.16	0.58	3.33	1.00	1.33	4.00	0.00	9.89
	Nurse-Employee	3.43	0.53	3.67	1.00	1.33	4.00	0.00	28.14
6	**Emotional dissonance**	**2.94**	**0.68**	**3.00**	**1.00**	**1.00**	**4.00**	**0.27**	**13.51**
	Doctor-Supervisor	2.73	0.68	2.67	0.67	1.00	4.00	1.33	8.00
	Doctor-Employee	2.80	0.59	2.67	0.83	2.00	4.00	0.00	8.11
	Nurse-Supervisor	2.96	0.67	3.00	0.67	1.33	4.00	0.00	14.29
	Nurse-Employee	3.05	0.70	3.00	1.00	1.33	4.00	0.00	16.77
***Work organisation***									
7	**Work time design**	**3.06**	**0.86**	**3.33**	**1.67**	**1.00**	**4.00**	**4.59**	**27.03**
	Doctor-Supervisor	2.32	0.90	2.33	1.33	1.00	4.00	18.67	6.67
	Doctor-Employee	3.07	0.84	3.33	1.00	1.00	4.00	2.70	16.22
	Nurse-Supervisor	2.92	0.75	3.00	1.33	1.00	4.00	2.20	17.58
	Nurse-Employee	3.46	0.64	3.67	1.00	1.67	4.00	0.00	43.71
8	**Overtime**	**2.88**	**0.79**	**3.00**	**1.33**	**1.00**	**4.00**	**1.62**	**14.86**
	Doctor-Supervisor	3.12	0.81	3.33	1.33	1.00	4.00	4.00	26.67
	Doctor-Employee	2.84	0.78	3.00	1.17	1.33	4.00	0.00	10.81
	Nurse-Supervisor	2.94	0.79	3.00	1.33	1.33	4.00	0.00	18.68
	Nurse-Employee	2.76	0.75	2.67	1.00	1.00	4.00	1.80	8.38
9	**Work intensity**	**3.48**	**0.60**	**3.67**	**1.00**	**1.00**	**4.00**	**0.27**	**43.24**
	Doctor-Supervisor	3.34	0.66	3.33	1.00	1.33	4.00	0.00	33.33
	Doctor-Employee	3.44	0.63	3.67	1.00	2.00	4.00	0.00	43.24
	Nurse-Supervisor	3.50	0.55	3.67	1.00	2.00	4.00	0.00	43.96
	Nurse-Employee	3.54	0.58	3.67	0.67	1.00	4.00	0.60	47.31
10	**Work interruptions**	**3.77**	**0.38**	**4.00**	**0.33**	**2.67**	**4.00**	**0.00**	**66.22**
	Doctor-Supervisor	3.70	0.44	4.00	0.67	2.67	4.00	0.00	62.67
	Doctor-Employee	3.81	0.37	4.00	0.17	3.00	4.00	0.00	75.68
	Nurse-Supervisor	3.71	0.39	4.00	0.33	2.67	4.00	0.00	54.95
	Nurse-Employee	3.82	0.35	4.00	0.33	2.67	4.00	0.00	71.86
**Violence Prevention Climate Scale (VPCS)** ^**d**^									
11	**Practices and responses**	**18.60**	**5.76**	**19.00**	**8.00**	**6.00**	**30.00**	**1.89**	**1.89**
	Doctor-Supervisor	21.32	5.24	21.00	7.00	7.00	30.00	0.00	2.67
	Doctor-Employee	18.65	5.97	20.00	7.50	6.00	29.00	2.70	0.00
	Nurse-Supervisor	20.37	5.13	20.00	6.00	7.00	30.00	0.00	3.30
	Nurse-Employee	16.41	5.44	16.00	8.00	6.00	30.00	3.59	1.20
12	**Policies and procedures**	**16.14**	**5.70**	**16.00**	**8.00**	**6.00**	**30.00**	**7.03**	**1.35**
	Doctor-Supervisor	16.97	5.98	18.00	8.00	6.00	30.00	8.00	2.67
	Doctor-Employee	14.97	5.05	15.00	8.50	6.00	24.00	8.11	0.00
	Nurse-Supervisor	18.54	4.92	19.00	7.00	7.00	30.00	0.00	1.10
	Nurse-Employee	14.72	5.65	15.00	9.00	6.00	30.00	10.18	1.20
13	**Pressure for unsafe practices**	**17.51**	**5.25**	**18.00**	**7.00**	**6.00**	**30.00**	**2.97**	**1.35**
	Doctor-Supervisor	15.15	4.93	16.00	7.00	6.00	27.00	4.00	0.00
	Doctor-Employee	17.30	4.56	18.00	6.50	10.00	30.00	0.00	2.70
	Nurse-Supervisor	16.15	5.37	17.00	8.00	6.00	28.00	6.59	0.00
	Nurse-Employee	19.35	4.84	20.00	6.00	6.00	30.00	1.20	2.40

^a^*N* = 370; doctor-supervisors (*n* = 75), doctor-employees (*n* = 37), nurse-supervisors (*n* = 91), and nurse-employees (*n* = 167)

^b^ Possible values for QPRA between 1–4 and for VPCS between 6–30

^c^ Floor- and ceiling-effects: the percentages of those achieving the lowest possible score (floor-effect) or the highest possible score (ceiling-effect) are displayed

^d^ Questionnaire for Psychosocial Risk Assessment (QPRA) according to Dettmers and Krause [13], and Violence Prevention Climate Scale (VPCS) according to Kessler et al. [21]

Descriptive statistics of the main study variables are displayed in Table 2 for the study sample as a whole and divided into groups as per profession and position in the ED.

On several subscales of the QPRA, high ceiling-effects were visible in the total sample or specific groups. In the undivided sample, four of the subscales showed ceiling-effects above 20%. Out of those, there were ceiling-effects above 40% on the subscale of work intensity, and above 65% on the subscale of work interruptions, respectively.

Spearman correlation coefficients were calculated (Table C, additional file 1). All three VPCS-dimensions showed significant correlations with most job demands and all job resources assessed by means of the QPRA. The two highest significant correlations of VPCS-dimensions with QPRA-subscales were observed between pressure for unsafe practices on the one hand, and one job resource (feedback and recognition, *r*_s_ = − 0.314, 95% BCa CI [-0.410, − 0.221], *p* < 0.001) as well as one job demand (social stressors, *r*_s_ = 0.306, 95% BCa CI [0.207, 0.411], *p* < 0.001) on the other hand. Furthermore, Table C (additional file 1) provides the values for Cronbach’s α, ranging from 0.743 to 0.903 and thus, indicating pertinent internal consistency for each (sub-)scale.

### Group comparisons for psychosocial working conditions and violence prevention climate

The four groups compared were doctor-supervisors, doctor-employees, nurse-supervisors, and nurse-employees (Table 3). A detailed overview of the follow-up analysis using pairwise comparisons can be found in Table D (additional file 1), the median is provided in Table 2. The effect sizes related to the significant pairwise comparisons were mostly small-medium or medium.

**Table 3:** Comparisons between groups according to profession and position along variables of QPRA and VPCS

Variables		Kruskal-Wallis-H	df	***p***-value Monte Carlo Significance^a^	99% CI^b^	Adjusted *p*-value^c^
**Questionnaire for Psychosocial Risk Assessment (QPRA)** ^**d**^						
***Social relations***						
1	Social support from colleagues	6.17	3	0.104	[0.101, 0.106]	0.208
2	Social support from supervisors	7.49	3	0.057	[0.056, 0.059]	0.230
3	Feedback and recognition	18.38	3	< 0.001	[0.000, 0.000]	**0.003**
4	Social stressors	6.82	3	0.076	[0.073, 0.078]	0.227
***Emotional load***						
5	Social and emotional demands	51.06	3	< 0.001	[0.000, 0.000]	**< 0.001**
6	Emotional dissonance	14.92	3	0.002	[0.001, 0.002]	**0.012**
***Work organisation***						
7	Work time design	87.69	3	< 0.001	[0.000, 0.000]	**< 0.001**
8	Overtime	12.84	3	0.005	[0.004, 0.005]	**0.029**
9	Work intensity	5.81	3	0.121	[0.119, 0.124]	0.121
10	Work interruptions	8.91	3	0.030	[0.029, 0.031]	0.150
**Violence Prevention Climate Scale (VPCS)** ^**d**^						
11	Practices and responses	49.31	3	< 0.001	[0.000, 0.000]	**< 0.001**
12	Policies and procedures	28.96	3	< 0.001	[0.000, 0.000]	**< 0.001**
13	Pressure for unsafe practices	41.19	3	< 0.001	[0.000, 0.000]	**< 0.001**

Groups compared: doctor-supervisors (*n* = 75), doctor-employees (*n* = 37), nurse-supervisors (*n* = 91), and nurse-employees (*n* = 167). Significant results (adjusted *p* ≤ 0.050) in bold

^a^ Based on 100,000 sampled tables

^b^ 99% CI given for unadjusted *p*-value (Monte Carlo Significance)

^c^ Holm-Bonferroni corrected across the 13 Kruskal-Wallis tests

^d^ Questionnaire for Psychosocial Risk Assessment (QPRA) according to Dettmers and Krause [13], and Violence Prevention Climate Scale (VPCS) according to Kessler et al. [21]

#### Psychosocial working conditions

##### Social relations

Concerning the QPRA-subscales on social relations, only the subscale on feedback and recognition was assessed significantly different between the aforementioned groups, *H*(3) = 18.38, *p* = 0.003. This result can be ascribed to only one significant difference in the pairwise comparisons, namely, between doctor-employees (Mdn = 3.00) and nurse-employees (Mdn = 2.33) with a small-medium effect size (*p* < 0.001, *r* = 0.283). Hence, only A1c could be supported, while the study failed to reject the null assumptions for A1a, A1b, and A1d (Table E, additional file 1).

##### Emotional load

Both of the QPRA-subscales on emotional load were rated significantly different between the groups, i.e., social and emotional demands: *H*(3) = 51.06, *p* < 0.001 and emotional dissonance: *H*(3) = 14.92, *p* = 0.012. For social and emotional demands, the nurse-employees, Mdn = 3.67, differed significantly from all other groups, i.e., the nurse-supervisors, Mdn = 3.33, (*p* < 0.001, *r* = − 0.221), doctor-supervisors, Mdn = 2.67, (*p* < 0.001, *r* = − 0.440), and the doctor-employees, Mdn = 3.00, (*p* = 0.004, *r* = − 0.232). In addition, the nurse-supervisors, Mdn = 3.33, differed significantly from the doctor-supervisors, Mdn = 2.67, (*p* = 0.006, *r* = − 0.244). Regarding emotional dissonance, the only significant pairwise comparison was between nurse-employees, Mdn = 3.00, and doctor-supervisors, Mdn = 2.67, (*p* < 0.001, *r* = − 0.227). In summary, A2a and A2b could be supported (Table E, additional file 1).

##### Work organisation

The Kruskal-Wallis tests on the QPRA-subscales on work organisation showed three significant results, but only two remained significant after correcting the *p*-values for multiple comparisons: firstly, the subscale assessing work time design, *H*(3) = 87.69, *p* < 0.001, and secondly, the subscale on overtime, *H*(3) = 12.84, *p* = 0.029. Regarding work time design, all pairwise comparisons were found to be significant, except the one between nurse-supervisors, Mdn = 3.00, and doctor-employees, Mdn = 3.33, (*p* = 0.281, *r* = 0.095). Additionally, across all follow-up tests, the only pairwise comparison showing a large effect size was found amongst those for work time design, i.e., between doctor-supervisors, Mdn = 2.33, and nurse-employees, Mdn = 3.67, (*p* < 0.001, *r* = − 0.581). Regarding the subscale for overtime, only one pairwise comparison was found to be significant, again between doctor-supervisors, Mdn = 3.33, and nurse-employees, Mdn = 2.67, (*p* < 0.001, *r* = 0.226). Regarding work organisation, A3a and A3b could be supported, while the study failed to reject the null assumptions for A3c and A3d (Table E, additional file 1).

#### Violence prevention climate

Regarding VPCS, all three scales were assessed significantly different by the groups (practices and responses: *H*(3) = 49.31, *p* < 0.001; policies and procedures: *H*(3) = 28.96, *p* < 0.001; pressure for unsafe practices: *H*(3) = 41.19, *p* < 0.001). The follow-up analysis showed several significant differences, e.g., between the nurse-employees and both groups of supervisors for all three VPCS-dimensions (Table D, additional file 1). The nurse-employees further differed significantly from the doctor-employees on the scales of practices and responses (*p* = 0.048, *r* = 0.177) and pressure for unsafe practices (*p* = 0.024, *r* = − 0.192). Here, the nurses reported significantly lower practices and responses towards violence prevention from the hospital management (nurse-employees: Mdn = 16.00; doctor-employees: Mdn = 20.00) as well as significantly higher pressure (nurse-employees: Mdn = 20.00; doctor-employees: Mdn = 18.00). In summary, A4a-A4c could be supported (Table E, additional file 1).

### Association of social relations with pressure for unsafe practices

In the first step of the hierarchical linear regression model, the indicator-coded control variable for belonging to groups according to profession and position was entered (Table 4, Model 1).

**Table 4:** Hierarchical linear regression model – predictors of pressure for unsafe practices

	Model 1				Model 2				Model 3				Model 4				
Variable	b [95% BCa CI]	SE	β	*p*	b [95% BCa CI]	SE	β	*p*	b [95% BCa CI]	SE	β	*p*	b [95% BCa CI]	SE	β	*p*	
Constant	15.15 [14.07, 16.28]	0.58		**0.001**	21.94 [18.44, 25.69]	1.71		**0.001**	22.28 [18.73, 26.06]	1.73		**0.001**	17.70 [13.12, 22.22]	2.42		**0.001**	
Doctor-Employee	2.15 [0.23, 4.00]	0.98	0.12	**0.022**	2.77 [0.85, 4.78]	1.01	0.16	**0.007**	2.91 [0.95, 4.86]	1.00	0.17	**0.002**	2.93 [1.04, 4.83]	0.97	0.17	**0.001**	
Nurse-Supervisor	1.01 [-0.52, 2.52]	0.81	0.08	0.229	1.44 [-0.03, 2.95]	0.78	0.12	0.062	1.33 [-0.07, 2.84]	0.77	0.11	0.075	1.13 [-0.32, 2.65]	0.80	0.09	0.161	
Nurse-Employee	4.21 [2.92, 5.46]	0.69	0.40	**0.001**	4.25 [2.95, 5.51]	0.69	0.40	**0.001**	4.08 [2.76, 5.36]	0.69	0.39	**0.001**	3.80 [2.33, 5.11]	0.73	0.36	**0.001**	
Social support from colleagues					-0.85 [-1.80, 0.09]	0.50	− 0.10	0.093	-0.66 [-1.60, 0.34]	0.50	− 0.08	0.201	-0.18 [-1.18, 0.94]	0.53	− 0.02	0.750	
Social support from supervisors					-1.48 [-2.15, -0.85]	0.34	− 0.23	**0.001**	-0.96 [-1.76, -0.24]	0.41	− 0.15	**0.023**	-0.88 [-1.69, -0.11]	0.42	− 0.14	**0.036**	
Feedback and recognition									-0.95 [-1.85, 0.01]	0.47	− 0.14	0.054	-0.85 [-1.81, 0.14]	0.47	− 0.13	0.076	
Social stressors													1.36 [0.36, 2.45]	0.49	0.17	**0.007**	
**Δ** ***R*** ^***2***^	0.113 **(*p*** **< 0.001)**				0.080 **(*p*** **< 0.001)**				0.010 **(*p*** **= 0.031)**				0.023 **(*p*** **= 0.001)**				
***R*** ^***2***^	0.113				0.194				0.204				0.227				
**Adjusted ** ***R*** ^***2***^	0.106				0.183				0.191				0.212				

*N* = 370; doctor-supervisors (*n* = 75), doctor-employees (*n* = 37), nurse-supervisors (*n* = 91), and nurse-employees (*n* = 167). Control variables were entered in model 1, with doctor-supervisors serving as reference group and thus, not being entered into the model. BCa CI, *p*-values, and SE based on 1,000 bootstrap samples. Significant results (*p* ≤ 0.050) in bold

In the second model, both social support from colleagues and from supervisors were added (Δ*R*^*2*^ = 0.080, *p* < 0.001) and the model significantly predicted pressure for unsafe practices, *F* (5, 364) = 17.48, *p* < 0.001 (Table 4, Model 2).

In the third block another job resource, i.e., feedback and recognition, was entered into the model, leading to an increment of 0.010 in *R*^*2*^ (*p* = 0.031). Once more, the model significantly predicted pressure for unsafe practices *F* (6, 363) = 15.50, *p* < 0.001 (Table 4, Model 3).

Lastly, in the fourth model, the variable of social stressors was included, leading to a significant change in *R*^*2*^ (Δ*R*^*2*^ = 0.023, *p* = 0.001). This final model was again able to significantly predict pressure for unsafe practices, *F* (7, 362) = 15.19, *p* < 0.001, and explained 21.2% of variance (Table 4, Model 4). For this final model, Cohen *f*^2^ = 0.269 was calculated, denoting a medium-large effect size.

**Fig. 2:**
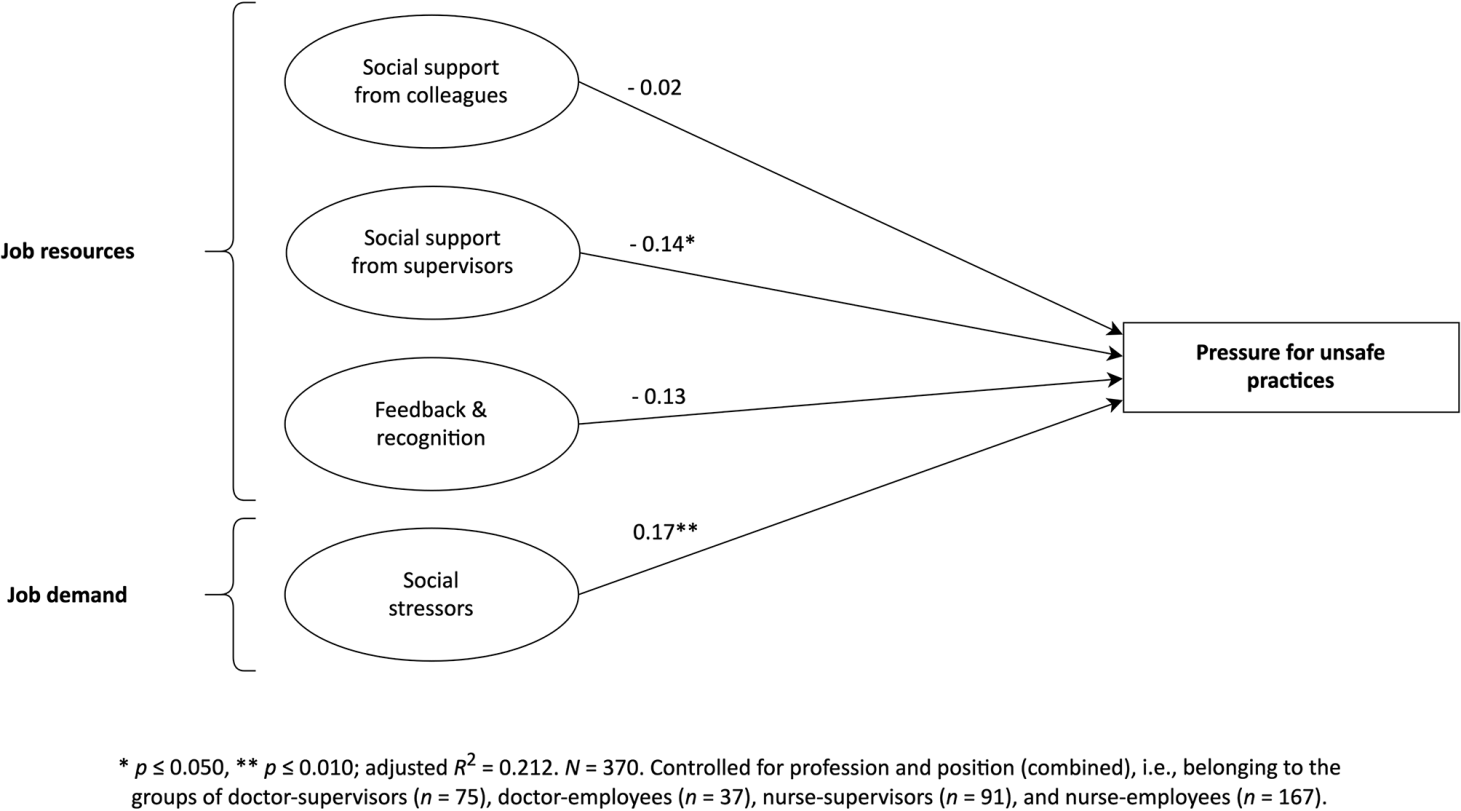
Conceptual model of the hierarchical multiple linear regression (final model) – predictors of pressure for unsafe practices with standardised beta-coefficients

Looking at the final model (Fig. 2), social support from supervisors (A5b) showed a significant negative relationship with pressure for unsafe practices (β = − 0.14, *p* = 0.036). However, the remaining two job resources in the model related to A5a and A5c, i.e., social support from colleagues (β = − 0.02, *p* = 0.750) as well as feedback and recognition (β = − 0.13, *p* = 0.076), were not significant in predicting pressure for unsafe practices. Finally, the job demand of social stressors (A5d) showed a significant positive relationship with pressure for unsafe practices (β = 0.17, *p* = 0.007). Therefore, A5b and A5d could be supported, while the study failed to reject the null assumptions for A5a and A5c (Table E, additional file 1).

#### Moderating role of profession combined with position

The relationships between the variables pertaining to social relations and pressure for unsafe practices were not significantly moderated by the variable defining membership in groups according to profession and position. Hence, none of the interactions between the grouping variable (moderator) and job resources pertaining to social relations (predictors) was significant in predicting pressure for unsafe practices (outcome), i.e., social support from colleagues (Δ*R²* = 0.004, *F*(3, 362) = 0.57, *p* = 0.636), social support from supervisors (Δ*R²* = 0.008, *F*(3, 362) = 0.81, *p* = 0.489), as well as feedback and recognition (Δ*R²* = 0.003, *F*(3, 362) = 0.37, *p* = 0.777). Likewise, belonging to a specific group regarding profession and position did not significantly moderate the relationship between the job demand of social stressors and pressure for unsafe practices (Δ*R²* = 0.008, *F*(3, 362) = 0.93, *p* = 0.429). Thus, the study failed to reject the null assumptions for A6a-d (Table E, additional file 1).

## Discussion

According to the aim of the study, perceptions regarding the violence prevention climate as well as psychosocial working conditions (i.e., job demands and resources) were compared among doctor-supervisors, doctor-employees, nurse-supervisors, and nurse-employees from German EDs. Furthermore, the associations between factors regarding social relations and pressure for unsafe practices were analysed, including a potential moderating role of the variable dividing the sample into four groups based on profession and position.

The first two research questions of this study were designed to find out whether the four groups have different perceptions concerning (a) job demands and resources related to psychosocial working conditions and (b) the violence prevention climate. The related assumptions were tested by means of Kruskal-Wallis tests. Eight out of 13 tests turned out to be significant after adjusting for multiple testing, indicating differences between the groups regarding both psychosocial working conditions and the violence prevention climate. Among the post-hoc pairwise comparisons, the highest number of significant differences was found between doctor-supervisors and nurse-employees, who differed on seven out of 13 variables. The smallest number of significant differences was found between doctor-supervisors and doctor-employees, as well as nurse-supervisors and doctor-employees: for both pairs a significant pairwise comparison could only be found for one variable. The effect sizes corresponding to significant pairwise comparisons were mostly small-medium or medium. One pairwise comparison regarding work time design showed a large effect size, i.e., between doctor-supervisors and nurse-employees, indicating a high practical significance of this result.

The third research question was formulated to find out if job demands and resources pertaining to social relations are associated with pressure for unsafe practices regarding violence prevention in German EDs. This was complemented by the fourth research question, asking if these relationships are moderated by belonging to a certain group in terms of profession and position. In line with the underlying framework of the JD-R model, the hierarchical multiple linear regression model revealed that one job resource (i.e., supervisor support) as well as one job demand (i.e., social stressors) were significant in predicting pressure for unsafe practices, while controlling for membership in groups according to profession and position. Furthermore, the effect size for the final model was medium-large, lending these results practical significance. However, none of the relationships between social relations and pressure for unsafe practices was significantly moderated by belonging to a certain group regarding profession and position.

### Group comparisons for psychosocial working conditions and violence prevention climate

Below, the results of group comparisons concerning psychosocial working conditions and violence prevention climate in German EDs will be discussed and compared with findings from previous literature.

#### Psychosocial working conditions

##### Social relations

Regarding the four subscales of social relations, only the subscale of feedback and recognition showed statistically significant differences between the groups. Here, the only significant pairwise comparison was observed between doctor-employees and nurse-employees, with the former reporting significantly higher feedback and recognition compared to the latter. Previous literature has suggested, that communication as means of feedback might be hindered by vertical (i.e., power and status) and horizontal (i.e., tasks within the ED) divisions [27]. Intra- and interdisciplinary pathways have been depicted with emergency physicians having more communication partners for giving and receiving feedback as compared to nurses [27]. Moreover, disparities regarding recognition between doctors and nurses in the hospital setting have been described [66, 67]. In the past, ED nurses have evolved to professionals with substantial expertise and education [68]. However, nurses have been described to perceive a discrepancy between hierarchy and competencies [66], and a lack of recognition has been found to be a main contributor to dissatisfaction at work for them [69]. These factors aid in explaining the significant differences regarding feedback and recognition found in the current study between doctor-employees reporting the highest, and nurse-employees showing the lowest mean rank and median.

The central tendencies of perceptions regarding support from supervisors and colleagues as well as social stressors were not significantly different between the four groups. This adds to the current landscape of literature, providing various findings regarding social relations of doctors and nurses in the ED [6, 7, 70] and limited research on ED leadership [30]. Thus, more research is needed to investigate social relations between doctors, nurses, and their supervisors in EDs.

##### Emotional load

For both subscales of emotional load, statistically significant differences in central tendencies between groups were observed for this sample. The lowest mean ranks were found for doctor-supervisors, while the highest were observed in nurse-employees, with the respective pairwise comparisons being statistically significant on both subscales. Mean rank and median were lower for both groups of doctors compared to nurses on the two subscales, indicating a tendency of more emotional load for nurses, although several related pairwise comparisons were not statistically significant.

On the subscale of social and emotional demands, ceiling-effects for nurse-employees were visible and they differed significantly from all other groups, indicating that they suffer from more emotionally draining situations as well as more aggressive and outrageous behaviour. ED nurses have been found to spend more time in direct patient contact compared to doctors [10], giving a potential reason for higher exposure to violence [71]. Supervisors, in turn, spend time with superordinate tasks to manage ED processes [72], also leading to less time in direct contact with patients. These factors contribute to a possible explanation for higher social and emotional demands in nurse-employees compared to the other groups in this sample. Likewise, in a study among ED nurses from South Korea, younger nurses, those with fewer experience in EDs, and those with lower position (general nurse vs. charge nurse) have been reported to be more exposed to violence [38].

Emotional labour has been described as an important part of nurses’ [73] and doctors’ [74] professions, as well as for healthcare workers in superordinate positions [45, 46]. In a previous cross-sectional study among healthcare workers from Hungary, nurses have reported significantly higher emotional dissonance compared to doctors [75]. Likewise, in the current study, nurse-employees reported significantly higher emotional dissonance compared to doctor-supervisors. Although all other pairwise comparisons on this subscale were insignificant after adjusting for multiple testing, the mean rank and median also indicate that both groups of nurses (supervisors and employees) reported higher emotional dissonance compared to the groups of doctors.

##### Work organisation

Regarding work organisation, after correcting for multiple comparisons, the subscales of work time design and overtime showed statistically significant differences in the central tendencies between the groups compared. The mean rank and median in this sample indicate that doctor-supervisors experience the least and nurse-employees the most demands regarding work time design (e.g., highly variable working hours and unfavourable shift work), with high ceiling-effects in the group of nurse-employees. In contrast to this, doctor-supervisors perceived the highest and nurse-employees the lowest demands regarding overtime according to mean rank and median. Previous literature has found shift work and overtime to be among the main factors contributing to poor working conditions in hospitals [76]. In terms of medical leadership in hospitals, a systematic review has found that doctor leaders felt overtime was a result of managerial work adding up on top of their clinical work [11], giving a possible explanation for the findings of the current study. Poor work time design in emergency medicine has been researched, for instance, related to shift work [77–79], which has been described as an important factor to be considered during risk assessments concerning occupational health and safety in EDs [80]. This study adds to the current body of literature with the finding that most of the groups according to profession and position significantly differ from each other regarding the perception of work time design in EDs, indicating the need for targeted interventions.

On the subscales of work intensity and work interruptions strong ceiling-effects were visible for all groups, and no statistically significant difference within these high ratings could be determined after adjusting for multiple comparisons. Hence, according to the high median across all groups, ED staff in the current sample seemed to be highly affected by work intensity and work interruptions, regardless of profession and position. These findings are in line with previous studies. In a survey conducted by the DGINA, 98.6% of the 362 participating hospitals have reported staff shortages in their EDs, contributing to an increasing burden in emergency care across Germany [81]. Likewise, in a literature review on the working environment in EDs, workload and time pressure have been named as major stressors [2]. Moreover, frequent work interruptions and multitasking have been well documented in the emergency medicine context [49, 82, 83].

#### Violence prevention climate

The participants predominantly rated the scales of the VPCS in the middle scale range, but with significant differences on all three dimensions. All pairwise comparisons looking at nurse-employees, significantly differentiated them from the other groups. The only exception was the scale of policies and procedures, where they only differed significantly from both groups of supervisors, but not from the doctor-employees. Hence, nurse-employees in this sample perceived the lowest practices and responses provided by the hospital management and the highest pressure to neglect violence prevention because of factors like staff shortage and work pressure. In addition, they reported fewer violence prevention policies and procedures available in their ward compared to both groups of supervisors.

To the authors’ best knowledge, the VPCS has not been applied to compare different professions and positions in German EDs until now [22]. Research on related concepts has found that nurses perceive a poorer safety climate [84], and higher occupational risks compared to doctors [55]. Moreover, several studies have found a larger proportion of nurses being exposed to violence compared to doctors [31–33]. The nursing staff needed in EDs to provide comprehensive care [81, 85], and to apply violence prevention measures [19], has been reported to be lacking. These factors can aid in explaining the results of the current study.

The current study also adds more nuances about the role of position to previous research, as both groups of supervisors had a more positive perception on all three dimensions of the VPCS compared to nurse-employees. As leaders are the ones creating climate [20], they might be prone to better perceptions of the violence prevention climate. This is further supported by research regarding safety climate, where those in higher positions have given more positive ratings of the climate [84]. In addition, similar to older ED staff reporting a higher level of confidence in managing violence [40], leaders might perceive less demand for practices and policies, as well as less pressure for unsafe practices regarding violence prevention. This could further be explained by different tasks, as leaders do not only work in direct patient care, but also take on managerial tasks [11, 12, 72].

### Association of social relations with pressure for unsafe practices and the moderating role of profession combined with position

There is still a scarceness of research on the violence prevention climate. However, the results of the current study are consistent with theoretical considerations provided by the original authors of the VPCS [21] and correspond to processes suggested by the JD-R model [23].

The important role of supervisors suggested by the authors of the VPCS [21] became clear in the results of the current study, as supervisor support was a significant negative predictor of pressure for unsafe practices, indicating a buffering effect of this job resource. Likewise, in previous research, supervisor and colleague support have been found to be significantly positively related to physical and psychosocial safety behaviour in healthcare workers [58].

The results further indicate that in comparison with colleagues’ support, in this sample, social stressors seemed to be more relevant: the latter turned out to be statistically significant as a positive predictor of pressure for unsafe practices, similar to the health impairment process of the JD-R model [23], while the former did not predict pressure.

In this sample, there seem to be limited differences between the four groups concerning social relations, as the majority of group comparisons and all four moderation models concerning these variables were statistically insignificant. On the other hand, measures regarding the violence prevention climate need to consider differences in groups’ perspectives that were visible in the group comparisons, and which also became apparent in the significant results for control variables in the hierarchical linear regression model.

### Strengths and limitations

The current study contributes to closing research gaps regarding psychosocial working conditions and the violence prevention climate in EDs, analysing them individually but also their association. To the authors’ best knowledge, this is the first research studying the violence prevention climate in German EDs [22]. Furthermore, this research considers both profession and position, taking the perspectives of doctors, nurses, and their respective supervisors within the ED into account.

Due to wide dispersion of the data and non-normal distribution, robust statistical methods were chosen like non-parametric tests, bootstrapping, the Monte Carlo method, or a heteroscedasticity-consistent standard error estimator. In addition, Holm-Bonferroni corrected *p*-values were reported to adjust alpha-levels for multiple comparisons. However, when interpreting the results several limitations need to be considered.

The cross-sectional study design is an important limitation, as it does not allow to infer causality. Regarding recruitment, efforts were taken in selecting EDs based on probability sampling. Furthermore, with support of the central directory of EDs [61], aiming to list all EDs in Germany, every ED was supposed to have equal chances to be contacted. Nevertheless, sampling bias might have been introduced, as only the medical and nursing leaders of EDs were contacted directly. The participation of employees relied on supervisors forwarding the questionnaire (i.e., snowball-sampling), and not all supervisors might support an assessment of working conditions or the violence prevention climate in their area of responsibility, potentially leading to non-response bias. However, the results do not suggest that only those giving favourable ratings participated, as for instance, high job demands were reported. In addition, a self-selection bias may have been present in the study due to voluntary participation, leading to those being more interested or having stronger opinions about the topic to participate and thus, potentially affecting the generalizability of results. Nevertheless, external validity of this study could be assumed, since participants were recruited from EDs all over Germany, and the sample consisted of a variety of participants with differing gender, age, position, and different levels of work experience. However, the sample might be biased by a relatively large proportion of participants coming from public hospitals and hospitals of G-BA level 3. To ensure anonymity of the respondents, hospital names were not acquired. Hence, the number of people participating per hospital could not be detected. Furthermore, response bias might have affected the results, as answering all questions was mandatory to prevent missing data, and due to the length of the questionnaire.

Furthermore, there were unequal group sizes, with nurse-employees alone making up around 45% of the sample. Considering the high nurse to doctor ratio in hospitals [86], the larger proportion of nurses compared to doctors in this sample could be plausible. However, the smallest group of doctor-employees only made up 10% of the sample, thus their perspectives might not have been fully captured and potential effects might not have been detected, limiting the generalisability of the results for their group. The unequal group sizes should be considered when interpreting the results. Several measures were taken to limit the impact of unequal group sizes. Before performing the Kruskal-Wallis test, homogeneity of variances between the groups was verified, and the Monte Carlo method was applied to obtain robust mean significance values and confidence intervals. In regression analysis, the variable defining membership in the four groups was included as control variable, and subsequent moderation analyses were performed and found to be insignificant. However, as explained in more detail in the methods section, the moderation analyses might have been impacted by a lack of linearity in two groups on the subscale of social support from colleagues and in one group on the subscale of feedback and recognition, which may be attributed to small group sizes and large dispersion of data points. Furthermore, when dealing with unequal groups sizes, it is suggested to use the effect size Cohen’s *d* instead of *r* [87]. However, as Cohen’s *d* requires normal distribution [88], which was also not given in the present dataset, *r* was used to report effect sizes for the pairwise comparisons, but should be interpreted cautiously.

Additional limitations need to be considered regarding the instruments used. The QPRA was applied in its validated German version [13], but high ceiling-effects in the total sample or specific groups were visible on several subscales, suggesting that in these cases the full range of employees’ perceptions might not have been captured. In addition, the VPCS had been checked for validity in its English version [21], but not in its German version translated in the course of this study. Furthermore, the QPRA was answered on a four-point Likert scale, while the VPCS was assessed on a five-point Likert scale. For both options, strengths and limitations need to be considered, e.g., participants’ attitudes might not be assessed correctly if a middle option is not available forcing them to choose an opinion, while on the other hand, the middle option might be disproportionally overused by participants [89].

The aforementioned ceiling-effects might have impacted the results of the Kruskal-Wallis test on the affected subscales. Here, the question arises whether the groups’ perceptions regarding these working conditions were correctly captured, or if in the upper scale range the instrument might not have been fully able to discern them. The latter might have led to potential differences not being detected or not appearing as strong as they might have actually been present in the sample. Likewise, the regression analysis might have been impacted by the ceiling-effects of approximately 20% on the subscale of social support from colleagues and approximately 15% on the subscale of social support from supervisors.

Lastly, there are potential confounding factors that were not considered in this study, like age, gender, or work experience. They were not included in the hierarchical linear regression model, as there are several detriments of including large numbers of control variables [64]. Since research on the violence prevention climate is still in its early stages [22], the main focus of this study lay on the different groups according to profession and position.

### Implications for practice

Several practical implications can be drawn from the current study, based on the differences and similarities between the groups. They should be taken into account when designing measures to improve psychosocial working conditions and the violence prevention climate in EDs.

Regarding work organisation, the participants perceived high levels of work intensity and work interruptions, which became apparent in ceiling-effects. Here, the massive burden on EDs becomes visible, which has been reported for EDs throughout Germany, with staff shortages being an important contributor [81]. In this regard, the legal requirement to guarantee acute care for emergency patients must be supported by the legislators, by allocating sufficient resources for emergency care [81] and by implementing recommendations for required staffing in EDs [85]. Factors related to the work environment, like adequate nurse staffing, have been previously discussed in relation to patient safety [90, 91], but need to be equally considered in terms of occupational safety for ED staff. Furthermore, work time design needs to be improved regarding highly variable working hours and unfavourable shift work – especially for nurse-employees. This can be done by implementing innovative concepts for work time design that are currently being tested [92].

Concerning social relations in EDs, feedback and recognition need to be improved, especially for nurse-employees, as they reported significantly lower levels compared to doctor-employees. This might not solely be the employers’ responsibility, but – as previous literature suggests – also a matter of social standing and equal appreciation for the professional groups of doctors and nurses [66].

When it comes to emotional load (e.g., dealing with aggressive and outrageous behaviour), nurse-employees reported the highest social and emotional demands according to mean rank and median. Likewise, they gave the most negative ratings on all dimensions of the VPCS according to the mean rank. So far, there is only little evidence concerning interventions for workplace violence prevention in EDs. A systematic review has shown that there are first results on multicomponent programmes (combining behavioural, organisational, and environmental interventions), but the majority of available research focuses on behavioural measures [93]. Furthermore, research on interventions targeted at the violence prevention climate in EDs is lacking. However, when it comes to explaining variance in safety outcomes, a meta-analytic investigation has found a supportive environment to be the most consistent job resource, as well as risks and hazards to be the most consistent job demand across industries [24]. Thus, conducting risk assessments and creating a supportive environment could help organisations to build a safe workplace and to increase the motivation of employees, e.g., by providing leadership training for supervisors, reinforcing teamwork, social support, and the value of safety [24].

In terms of violence prevention, this means awareness should be created at all levels including hospital management and supervisory positions. Interprofessional efforts should be promoted to create a supportive environment and distribute the responsibility of dealing with aggressive behaviour evenly – in order to disburden the colleagues being most affected. Furthermore, those in leadership positions need to consider the role of supervisor support in decreasing pressure for unsafe practices. They should act as role models and place the safety of their employees above productivity [21]. Peer support and measures related to teamwork in violence prevention have previously been implemented as part of a successful multicomponent intervention to reduce physical assaults in a psychiatric emergency room [94]. According to this train of thought, the results of the current study indicate that sorting out social stressors within the team could contribute to decreasing pressure for unsafe practices, which for instance, could be supported by regular team supervisions.

### Implications for future research

This research found that there are significant differences between the groups of doctors, nurses, and their respective supervisors within the ED regarding psychosocial working conditions and the violence prevention climate. However, due to the limitations discussed above, further research is needed to verify if these results can be confirmed. In this context, special emphasis during recruitment should be put on the group of doctor-employees, as they were the smallest group in this sample and their opinions might not have been fully captured.

Nursing and physician leadership have often been studied independently from one another [11, 12], and there is limited literature on differences and similarities between nurses and doctors as ED leaders [72]. This limited knowledge needs to be further expanded to find out if both leadership groups can benefit from similar support systems.

High ceiling-effects became apparent using the QPRA for assessing psychosocial working conditions. Hence, future research needs to either adapt and refine this instrument, or apply different ones, e.g., [95]. In addition, there is a scarcity of research on the violence prevention climate [22], and future research needs to assess if interventions targeted at the violence prevention climate can improve safety outcomes related to violence. Future research can also analyse potential confounding factors like gender, which has been reported to be relevant concerning violence in EDs [40, 96].

## Conclusion

The present study expands the current body of literature in several ways. To the authors’ best knowledge, this is the first study in an ED setting not only comparing doctors and nurses but also their respective supervisors regarding psychosocial working conditions and the violence prevention climate. In addition, it is also analysing associations between factors of social relations with pressure for unsafe practices regarding violence prevention, and the moderating role of belonging to a certain group regarding profession and position in these relationships.

The results suggest that there are several important differences between the groups of doctor-supervisors, doctor-employees, nurse-supervisors, and nurse-employees within the ED. These differences should be considered when designing measures for occupational health and safety. For instance, nurse-employees reported the highest social and emotional demands as well as the most pressure for unsafe practices, while perceiving the lowest practices and responses regarding violence prevention, differing significantly from all other groups on the respective variables. Furthermore, ED staff reported high levels of work intensity and work interruptions, which need to be prioritised to improve the working conditions, e.g., by providing adequate resources and staffing for EDs.

With the goal of decreasing pressure for unsafe practices regarding violence prevention, supervisors’ support for ED staff should be strengthened and interprofessional support within the team should be reinforced to limit social stressors. Joint efforts by the hospital management as well as leaders and employees are needed to improve the working conditions and the violence prevention climate in German EDs, with the help of measures tailored according to the needs of different professions and positions.

## Electronic Supplementary Material

Below is the link to the electronic supplementary material.

**Supplementary Material 1**: **Additional File 1**: Psychosocial Working Conditions and Violence Prevention Climate in German Emergency Departments – A Cross-Sectional Study

**Supplementary Material 1**: **Additional File 2**: STROBE Statement

## Data Availability

The datasets generated and analysed during the current study are not publicly available due to German national data protection regulations. They are available from the corresponding author on reasonable request.

## Abbreviations

BCa CI: Bias-corrected and accelerated confidence interval(s)
DGINA: German Society for Interdisciplinary Emergency and Acute Medicine. (German: Deutsche Gesellschaft Interdisziplinäre Notfall- und Akutmedizin)
DIVI: German Interdisciplinary Association for Intensive and Emergency Care. (German: Deutsche Interdisziplinäre Vereinigung für Intensiv- und Notfallmedizin)
ED(s): Emergency department(s)
G-BA: Federal Joint Committee (German: Gemeinsamer Bundesausschuss)
ILO: International Labour Organization
JD-R model: Job Demands-Job Resources model
LOESS: Locally estimated scatterplot smoothing
QPRA: Questionnaire for Psychosocial Risk Assessment (German: FGBU = Fragebogen zur Gefährdungsbeurteilung psychischer Belastungen)
STROBE: Strengthening the Reporting of Observational Studies in Epidemiology
VIF: Variance inflation factor
VPCS: Violence Prevention Climate Scale
