# Supplementary material for: Psychosocial working conditions and violence prevention climate in German emergency departments – a cross-sectional study
Source: BMC Emerg Med. 2025 Jan 23;25:17. doi: 10.1186/s12873-024-01155-y (PMC11759433; doi:10.1186/s12873-024-01155-y)
Supplement: Supplementary file 1 — Supplementary Material 1: Additional File 1: Psychosocial Working Conditions and Violence Prevention Climate in German Emergency Departments – A Cross-Sectional Study [file 12873_2024_1155_MOESM1_ESM.pdf]

Additional File 1

Psychosocial Working Conditions and Violence Prevention Climate in German Emergency Departments – A Cross-Sectional Study

Sonja REIßMANN[1,2], Mannat GULIANI[1], Tanja WIRTH[1], David A. GRONEBERG[2], Volker HARTH[1] and Stefanie MACHE[1,2*]

[1] Institute for Occupational and Maritime Medicine (ZfAM), University Medical Center Hamburg-Eppendorf (UKE), 20459 Hamburg, Germany.

[2] Institute of Occupational Medicine, Social Medicine and Environmental Medicine, Goethe University Frankfurt, 60590 Frankfurt, Germany.

List of Tables in the Additional File 1:

Table A: Overview of the questionnaire applied in the current study

Table B: Hospital characteristics as stated by participants (*N* = 370)

Table C: Spearman's correlation coefficients among the study variables

Table D: Pairwise comparisons conducted as follow-up analysis for Kruskal-Wallis tests

Table E: Overview of null assumption significance testing in the current study

Table A: Overview of the questionnaire applied in the current study

Section	Items / subscales / scales	Number of items
Inclusion criteria	Spatially separated ED, minimum of three months of work experience in the current ED, direct contact with patients, profession (doctor or nurse)	4
Participant and hospital characteristics	Gender, age, position (supervisor or employee), working hours, work experience in the current ED, total work experience in any ED, emergency care level of the ED, number of hospital beds, type of hospital funding, federal state in which the hospital is located	11
Questionnaire for Psychosocial Risk Assessment (QPRA)[a]	*Social relations*	
	Social support from colleagues	3
	Social support from supervisors	3
	Feedback and recognition	3
	Social stressors	3
	Emotional load	
	Social and emotional demands	3
	Emotional dissonance	3
	Work organisation	
	Work time design	3
	Overtime	3
	Work intensity	3
	Work interruptions	3
Health-oriented Leadership (HoL)[b]	HoL SelfCare (answered by supervisors and employees)	19
	HoL StaffCare: supervisors' assessment of their own staff-care (only answered by supervisors)	22 (only supervisors)
	HoL StaffCare: employees' assessment of their supervisors' staff-care (only answered by employees)	22 (only employees)
Violence Prevention Climate Scale (VPCS)[c]	Practices and responses	6
	Policies and procedures	6
	Pressure for unsafe practices	6
	Total number of items for each participant	104

ED = Emergency department

[a] According to Dettmers, J. and Krause, A., *Der Fragebogen zur Gefährdungsbeurteilung psychischer Belastungen (FGBU).* Zeitschrift für Arbeits- und Organisationspsychologie A&O, 2020. 64(2): 99-119. https://doi.org/10.1026/0932-4089/a000318.

[b] The HoL-scales were not considered for the current publication. However, analyses containing HoL-scales were already published: Guliani, M., et al., *Violence Prevention Climate and Health-Oriented Leadership in German Emergency Departments*. Healthcare, 2023. 11(16): 2234. https://doi.org/10.3390/healthcare11162234.

[c] According to Kessler, S. R., et al., *Organizational violence and aggression: Development of the three-factor Violence Climate Survey.* Work & Stress, 2008. 22(2): 108-124. https://doi.org/10.1080/02678370802187926.

Table B: Hospital characteristics as stated by participants (*N* = 370)

**Variables**		***n***	**%**
Emergency care level of the ED[a]	G-BA Level 3	160	43.2
	G-BA Level 2	103	27.8
	G-BA Level 1	81	21.9
	G-BA Level 0[b]	11	3.0
	Unknown	15	4.1
Number of hospital beds	≥600	141	38.1
	300-599	140	37.8
	≤299	76	20.5
	Unknown	13	3.5
Type of hospital funding	Commercial (profit-oriented)	63	17.0
	Public	211	57.0
	Independent (non-profit, charity, church)	89	24.1
	Unknown	7	1.9
Federal state in which the hospital is located	Baden-Württemberg	48	13.0
	Bavaria	53	14.3
	Berlin	20	5.4
	Brandenburg	4	1.1
	Bremen	14	3.8
	Hamburg	9	2.4
	Hesse	26	7.0
	Mecklenburg-Western Pomerania	6	1.6
	Lower Saxony	35	9.5
	North Rhine-Westphalia	68	18.4
	Rhineland-Palatinate	19	5.1
	Saarland	3	0.8
	Saxony	8	2.2
	Saxony-Anhalt	9	2.4
	Schleswig-Holstein	36	9.7
	Thuringia	12	3.2

ED = Emergency department, G-BA = Gemeinsamer Bundesausschuss (English: Federal Joint Committee)

[a] According to the Federal Joint Committee: GKV-Spitzenverband. Prognose der Notfallstufen nach § 136c Absatz 4 SGB V, Stand 12.05.2021. 2021. [cited: 05.07.2024]; Available from: https://www.gkv-spitzenverband.de/media/dokumente/krankenversicherung_1/krankenhaeuser/KH_Corona_Final_Prognose_Notfallstufen_Stand_12-05-2021_barrierefrei.pdf.

[b] In accordance with the eligibility criteria, hospitals with G-BA level 0 were not contacted during the first stage of recruitment, because they may not have a spatially separated emergency ward. However, if participants working in a hospital with G-BA level 0 fulfilled the inclusion criterion of working in a spatially separated emergency ward, they were retained in the sample.

Table C: Spearman's correlation coefficients among the study variables

Variable	1	2	3	4	5	6	7	8	9	10	11	12	13
**1 Social support from colleagues**	(.853)												
**2 Social support from supervisors**	.368***<br>[.277, .458]	(.903)											
**3 Feedback and recognition**	.374***<br>[.279, .469]	.667***<br>[.598, .727]	(.840)										
**4 Social stressors**	-.416***<br>[-.508, -.331]	-.271***<br>[-.364, -.162]	-.298***<br>[-.390, -.206]	(.805)									
**5 Social and emotional demands**	.005<br>[-.096, .106]	-.055<br>[-.154, .039]	-.135**<br>[-.238, -.039]	.256***<br>[.155, .349]	(.743)								
**6 Emotional dissonance**	-.010<br>[-.111, .085]	-.098<br>[-.201, .001]	-.097<br>[-.199, .009]	.180***<br>[.068, .285]	.497***<br>[.408, .579]	(.780)							
**7 Work time design**	-.052<br>[-.154, .053]	-.158**<br>[-.260, -.060]	-.211***<br>[-.307, -.120]	.142**<br>[.034, .251]	.426***<br>[.334, .510]	.252***<br>[.137, .345]	(.848)						
**8 Overtime**	-.095<br>[-.208, .016]	-.175***<br>[-.276, -.074]	-.048<br>[-.160, .068]	.236***<br>[.134, .330]	.164**<br>[.067, .257]	.209***<br>[.108, .306]	.142**<br>[.047, .237]	(.770)					
**9 Work intensity**	-.058<br>[-.161, .054]	-.190***<br>[-.283, -.088]	-.169***<br>[-.278, -.054]	.236***<br>[.146, .316]	.354***<br>[.260, .442]	.312***<br>[.216, .406]	.321***<br>[.230, .416]	.511***<br>[.429, .580]	(.837)				
**10 Work interruptions**	-.102<br>[-.209, .008]	-.105*<br>[-.198, .000]	-.126*<br>[-.222, -.024]	.258***<br>[.165, .347]	.252***<br>[.144, .352]	.253***<br>[.155, .345]	.255***<br>[.160, .348]	.345***<br>[.255, .425]	.535***<br>[.447, .616]	(.797)			
**11 Practices and responses**	.132*<br>[.029, .233]	.185***<br>[.089, .278]	.285***<br>[.184, .375]	-.191***<br>[-.282, -.096]	-.235***<br>[-.327, -.146]	-.184***<br>[-.285, -.090]	-.256***<br>[-.348, -.157]	-.092<br>[-.207, .024]	-.221***<br>[-.324, -.120]	-.218***<br>[-.315, -.128]	(.885)		
**12 Policies and procedures**	.174***<br>[.069, .275]	.198***<br>[.101, .299]	.234***<br>[.117, .332]	-.139**<br>[-.245, -.035]	-.112*<br>[-.204, -.026]	-.135**<br>[-.236, -.037]	-.207***<br>[-.308, -.112]	-.045<br>[-.154, .072]	-.150**<br>[-.264, -.046]	-.111*<br>[-.208, -.013]	.589***<br>[.511, .662]	(.903)	
**13 Pressure for unsafe practices**	-.228***<br>[-.330, -.118]	-.263***<br>[-.361, -.167]	-.314***<br>[-.410, -.221]	.306***<br>[.207, .411]	.276***<br>[.183, .377]	.184***<br>[.078, .292]	.283***<br>[.179, .392]	.146**<br>[.042, .236]	.281***<br>[.191, .371]	.213***<br>[.121, .311]	-.504***<br>[-.580, -.421]	-.443***<br>[-.530, -.353]	(.894)

N = 370. Spearman correlation coefficient: * $p \leq .050$; ** $p \leq .010$; *** $p \leq .001$ (two-tailed). 95% bias-corrected and accelerated (BCa) confidence intervals (CI) reported in square brackets (based on 1,000 bootstrap samples). Cronbach's α in parentheses provided along the diagonal line.

Table D: Pairwise comparisons conducted as follow-up analysis for Kruskal-Wallis tests

	**Pairwise comparisons per variable[a,b]**	***p*-value[c]**	**Adjusted *p*-value[d]**	**Effect size (*r*)[e]**
**Questionnaire for Psychosocial Risk Assessment (QPRA)[f]**				
**Social relations**				
1	**Social support from colleagues:** $H(3) = 6.17$, $p = .208$<br>Mean rank: doc-sup = 164.32; doc-emp = 186.34; nur-sup = 204.52; nur-emp = 184.46			
	Doctor-Supervisor vs. Nurse-Supervisor	.013	.078	-.192
	Doctor-Supervisor vs. Doctor-Employee	.292	.876	-.100
	Doctor-Supervisor vs. Nurse-Employee	.163	.652	-.090
	Nurse-Supervisor vs. Doctor-Employee	.370	.740	-.079
	Nurse-Supervisor vs. Nurse-Employee	.139	.695	.092
	Nurse-Employee vs. Doctor-Employee	.921	.921	.007
2	**Social support from supervisors:** $H(3) = 7.49$, $p = .230$<br>Mean rank: doc-sup = 177.61; doc-emp = 222.80; nur-sup = 195.94; nur-emp = 175.09			
	Doctor-Supervisor vs. Nurse-Supervisor	.268	.536	-.086
	Doctor-Supervisor vs. Doctor-Employee	.034	.170	-.201
	Doctor-Supervisor vs. Nurse-Employee	.864	.864	.011
	Nurse-Supervisor vs. Doctor-Employee	.194	.582	.115
	Nurse-Supervisor vs. Nurse-Employee	.131	.524	.094
	Nurse-Employee vs. Doctor-Employee	.013	.078	.173
3	**Feedback and recognition:** $H(3) = 18.38$, $\boldsymbol{p = .003}$<br>Mean rank: doc-sup = 191.09; doc-emp = 242.49; nur-sup = 196.21; nur-emp = 164.52			
	Doctor-Supervisor vs. Nurse-Supervisor	.757	.757	-.024
	Doctor-Supervisor vs. Doctor-Employee	.016	.080	-.228
	Doctor-Supervisor vs. Nurse-Employee	.071	.142	.116
	Nurse-Supervisor vs. Doctor-Employee	.025	.075	.198
	Nurse-Supervisor vs. Nurse-Employee	.022	.088	.143
	Nurse-Employee vs. Doctor-Employee	< .001	**< .001**	.283
4	**Social stressors:** $H(3) = 6.82$, $p = .227$<br>Mean rank: doc-sup = 173.25; doc-emp = 160.64; nur-sup = 178.40; nur-emp = 200.38			
	Doctor-Supervisor vs. Nurse-Supervisor	.754	.754	-.024
	Doctor-Supervisor vs. Doctor-Employee	.551	1.000	.056
	Doctor-Supervisor vs. Nurse-Employee	.064	.320	-.119
	Nurse-Supervisor vs. Doctor-Employee	.387	1.000	-.076
	Nurse-Supervisor vs. Nurse-Employee	.109	.436	-.100
	Nurse-Employee vs. Doctor-Employee	.038	.228	-.145
**Emotional load**				
5	**Social and emotional demands:** $H(3) = 51.06$, $\boldsymbol{p < .001}$<br>Mean rank: doc-sup = 123.74; doc-emp = 160.59; nur-sup = 175.51; nur-emp = 224.20			
	Doctor-Supervisor vs. Nurse-Supervisor	.002	**.006**	-.244
	Doctor-Supervisor vs. Doctor-Employee	.082	.164	-.164
	Doctor-Supervisor vs. Nurse-Employee	< .001	**< .001**	-.440
	Nurse-Supervisor vs. Doctor-Employee	.468	.468	-.064
	Nurse-Supervisor vs. Nurse-Employee	< .001	**< .001**	-.221
	Nurse-Employee vs. Doctor-Employee	.001	**.004**	-.232
6	**Emotional dissonance:** $H(3) = 14.92$, $\boldsymbol{p = .012}$<br>Mean rank: doc-sup = 152.48; doc-emp = 159.70; nur-sup = 188.64; nur-emp = 204.33			
	Doctor-Supervisor vs. Nurse-Supervisor	.028	.112	-.170

	Doctor-Supervisor vs. Doctor-Employee	.734	.734	-.032
	Doctor-Supervisor vs. Nurse-Employee	< .001	**< .001**	-.227
	Nurse-Supervisor vs. Doctor-Employee	.160	.480	-.124
	Nurse-Supervisor vs. Nurse-Employee	.254	.508	-.071
	Nurse-Employee vs. Doctor-Employee	.020	.100	-.163
**Work organisation**				
7	**Work time design:** $H(3) = 87.69$, $\boldsymbol{p < .001}$<br>Mean rank: doc-sup = 102.87; doc-emp = 184.65; nur-sup = 162.48; nur-emp = 235.34			
	Doctor-Supervisor vs. Nurse-Supervisor	< .001	**< .001**	-.281
	Doctor-Supervisor vs. Doctor-Employee	< .001	**< .001**	-.365
	Doctor-Supervisor vs. Nurse-Employee	< .001	**< .001**	-.581
	Nurse-Supervisor vs. Doctor-Employee	.281	.281	.095
	Nurse-Supervisor vs. Nurse-Employee	< .001	**< .001**	-.330
	Nurse-Employee vs. Doctor-Employee	.008	**.016**	-.185
8	**Overtime:** $H(3) = 12.84$, $\boldsymbol{p = .029}$<br>Mean rank: doc-sup = 219.97; doc-emp = 179.62; nur-sup = 191.51; nur-emp = 168.05			
	Doctor-Supervisor vs. Nurse-Supervisor	.085	.340	.134
	Doctor-Supervisor vs. Doctor-Employee	.058	.290	.179
	Doctor-Supervisor vs. Nurse-Employee	< .001	**< .001**	.226
	Nurse-Supervisor vs. Doctor-Employee	.566	.566	-.051
	Nurse-Supervisor vs. Nurse-Employee	.090	.270	.106
	Nurse-Employee vs. Doctor-Employee	.548	1.000	.042
9	**Work intensity:** $H(3) = 5.81$, $p = .121$<br>Mean rank: doc-sup = 162.41; doc-emp = 180.05; nur-sup = 187.14; nur-emp = 196.18			
	Doctor-Supervisor vs. Nurse-Supervisor	.120	.600	-.121
	Doctor-Supervisor vs. Doctor-Employee	.389	1.000	-.081
	Doctor-Supervisor vs. Nurse-Employee	.017	.102	-.153
	Nurse-Supervisor vs. Doctor-Employee	.722	.722	-.031
	Nurse-Supervisor vs. Nurse-Employee	.496	.992	-.042
	Nurse-Employee vs. Doctor-Employee	.384	1.000	-.061
10	**Work interruptions:** $H(3) = 8.91$, $p = .150$<br>Mean rank: doc-sup = 174.38; doc-emp = 200.73; nur-sup = 167.00; nur-emp = 197.20			
	Doctor-Supervisor vs. Nurse-Supervisor	.598	1.000	.041
	Doctor-Supervisor vs. Doctor-Employee	.144	.432	-.138
	Doctor-Supervisor vs. Nurse-Employee	.068	.272	-.118
	Nurse-Supervisor vs. Doctor-Employee	.054	.270	.170
	Nurse-Supervisor vs. Nurse-Employee	.010	.060	-.161
	Nurse-Employee vs. Doctor-Employee	.829	.829	.015
**Violence Prevention Climate Scale (VPCS)[g]**				
11	**Practices and responses:** $H(3) = 49.31$, $\boldsymbol{p < .001}$<br>Mean rank: doc-sup = 235.03; doc-emp = 193.24; nur-sup = 217.24; nur-emp = 144.24			
	Doctor-Supervisor vs. Nurse-Supervisor	.285	.285	.083
	Doctor-Supervisor vs. Doctor-Employee	.051	.153	.184
	Doctor-Supervisor vs. Nurse-Employee	< .001	**< .001**	.393
	Nurse-Supervisor vs. Doctor-Employee	.249	.498	-.102
	Nurse-Supervisor vs. Nurse-Employee	< .001	**< .001**	.327
	Nurse-Employee vs. Doctor-Employee	.012	**.048**	.177
12	**Policies and procedures:** $H(3) = 28.96$, $\boldsymbol{p < .001}$<br>Mean rank: doc-sup = 200.47; doc-emp = 164.85; nur-sup = 230.08; nur-emp = 159.06			
	Doctor-Supervisor vs. Nurse-Supervisor	.075	.225	-.138
	Doctor-Supervisor vs. Doctor-Employee	.097	.194	.157

	Doctor-Supervisor vs. Nurse-Employee	.005	**.020**	.179
	Nurse-Supervisor vs. Doctor-Employee	.002	**.010**	-.277
	Nurse-Supervisor vs. Nurse-Employee	< .001	**< .001**	.318
	Nurse-Employee vs. Doctor-Employee	.765	.765	.021
13	**Pressure for unsafe practices:** $H(3) = 41.19$, $\boldsymbol{p < .001}$ Mean rank: doc-sup = 137.90; doc-emp = 170.18; nur-sup = 161.64; nur-emp = 223.28			
	Doctor-Supervisor vs. Nurse-Supervisor	.154	.308	-.111
	Doctor-Supervisor vs. Doctor-Employee	.132	.396	-.142
	Doctor-Supervisor vs. Nurse-Employee	< .001	**< .001**	-.370
	Nurse-Supervisor vs. Doctor-Employee	.681	.681	.036
	Nurse-Supervisor vs. Nurse-Employee	< .001	**< .001**	-.276
	Nurse-Employee vs. Doctor-Employee	.006	**.024**	-.192

Groups compared: doctor-supervisors (n = 75), doctor-employees (n = 37), nurse-supervisors (n = 91), and nurse-employees (n = 167). Significant results (adjusted $p \le .050$) in bold.

[a] Result of Kruskal-Wallis test and mean rank per group provided behind variable names.

[b] Median and interquartile range are provided in Table 2 (in the manuscript).

[c] Two-tailed significance values were obtained.

[d] Holm-Bonferroni correction performed for the six pairwise comparisons per variable.

[e] Pearson's correlation coefficient, with $r = .10$ indicating a small, $r = .30$ denoting a medium, and $r = .50$ suggesting a large effect according to Cohen: Cohen, J., Statistical Power Analysis for the Behavioral Sciences. 2nd ed. 1988, Lawrence Erlbaum Associates: Hillsdale, New Jersey.

[f] According to Dettmers, J. and Krause, A., *Der Fragebogen zur Gefährdungsbeurteilung psychischer Belastungen (FGBU).* Zeitschrift für Arbeits- und Organisationspsychologie A&O, 2020. 64(2): 99-119. https://doi.org/10.1026/0932-4089/a000318.

[g] According to Kessler, S. R., et al., *Organizational violence and aggression: Development of the three-factor Violence Climate Survey.* Work & Stress, 2008. 22(2): 108-124. https://doi.org/10.1080/02678370802187926.

Table E: Overview of null assumption significance testing in the current study

Assumption		Result
A1: Social relations (QPRA)[a]	A1a Social support from colleagues	Failed to reject null assumption
	A1b Social support from supervisors	Failed to reject null assumption
	**A1c Feedback and recognition**	**Alternative assumption supported**
	A1d Social stressors	Failed to reject null assumption
A2: Emotional load (QPRA)[a]	**A2a Social and emotional demands**	**Alternative assumption supported**
	**A2b Emotional dissonance**	**Alternative assumption supported**
A3: Work organisation (QPRA)[a]	**A3a Work time design**	**Alternative assumption supported**
	**A3b Overtime**	**Alternative assumption supported**
	A3c Work intensity	Failed to reject null assumption
	A3d Work interruptions	Failed to reject null assumption
A4: Violence Prevention Climate Scale (VPCS)[b]	**A4a Practices and responses**	**Alternative assumption supported**
	**A4b Policies and procedures**	**Alternative assumption supported**
	**A4c Pressure for unsafe practices**	**Alternative assumption supported**
A5: Associations of social relations with pressure for unsafe practices	A5a Association of social support from colleagues with pressure	Failed to reject null assumption
	**A5b Association of social support from supervisors with pressure**	**Alternative assumption supported**
	A5c Association of feedback and recognition with pressure	Failed to reject null assumption
	**A5d Association of social stressors with pressure**	**Alternative assumption supported**
A6: Moderating role of belonging to a group according to profession and position in the associations of social relations with pressure for unsafe practices	A6a Moderation in the relationship between social support from colleagues and pressure	Failed to reject null assumption
	A6b Moderation in the relationship between social support from supervisors and pressure	Failed to reject null assumption
	A6c Moderation in the relationship between feedback & recognition and pressure	Failed to reject null assumption
	A6d Moderation in the relationship between social stressors and pressure	Failed to reject null assumption

[a] Questionnaire for Psychosocial Risk Assessment (QPRA) according to Dettmers, J. and Krause, A., *Der Fragebogen zur Gefährdungsbeurteilung psychischer Belastungen (FGBU).* Zeitschrift für Arbeits- und Organisationspsychologie A&O, 2020. 64(2): 99-119. https://doi.org/10.1026/0932-4089/a000318.

[b] According to Kessler, S. R., et al., *Organizational violence and aggression: Development of the three-factor Violence Climate Survey.* Work & Stress, 2008. 22(2): 108-124. https://doi.org/10.1080/02678370802187926.
