# Supplementary material for: Psychosocial working conditions and violence prevention climate in German emergency departments – a cross-sectional study
Source: BMC Emerg Med. 2025 Jan 23;25:17. doi: 10.1186/s12873-024-01155-y (PMC11759433; doi:10.1186/s12873-024-01155-y)
Supplement: Supplementary file 2 — Supplementary Material 1: Additional File 2: STROBE Statement [file 12873_2024_1155_MOESM2_ESM.pdf]

STROBE Statement—Checklist of items that should be included in reports of ***cross-sectional studies***

	Item No	Recommendation	Section of the Manuscript
**Title and abstract**	1	(*a*) Indicate the study's design with a commonly used term in the title or the abstract	Title
		(*b*) Provide in the abstract an informative and balanced summary of what was done and what was found	Abstract
**Introduction**			
Background/rationale	2	Explain the scientific background and rationale for the investigation being reported	Background
Objectives	3	State specific objectives, including any prespecified hypotheses	Current State of Research
**Methods**			
Study design	4	Present key elements of study design early in the paper	Study Design
Setting	5	Describe the setting, locations, and relevant dates, including periods of recruitment, exposure, follow-up, and data collection	Study Design, Participants and Recruitment
Participants	6	(*a*) Give the eligibility criteria, and the sources and methods of selection of participants	Participants and Recruitment
Variables	7	Clearly define all outcomes, exposures, predictors, potential confounders, and effect modifiers. Give diagnostic criteria, if applicable	Variables and Measurement, Statistical Methods
Data sources/ measurement	8*	For each variable of interest, give sources of data and details of methods of assessment (measurement). Describe comparability of assessment methods if there is more than one group	Variables and Measurement
Bias	9	Describe any efforts to address potential sources of bias	Statistical Methods, Strengths and Limitations
Study size	10	Explain how the study size was arrived at	Participants and Recruitment, Statistical Methods
Quantitative variables	11	Explain how quantitative variables were handled in the analyses. If applicable, describe which groupings were chosen and why	Variables and Measurement, Statistical Methods
Statistical methods	12	(*a*) Describe all statistical methods, including those used to control for confounding	Statistical Methods
		(*b*) Describe any methods used to examine subgroups and interactions	Statistical Methods
		(*c*) Explain how missing data were addressed	Variables and Measurement
		(*d*) If applicable, describe analytical methods taking account of sampling strategy	n/a
		(*e*) Describe any sensitivity analyses	n/a

**Results**			
Participants	13*	(a) Report numbers of individuals at each stage of study—eg numbers potentially eligible, examined for eligibility, confirmed eligible, included in the study, completing follow-up, and analysed	Statistical Methods
		(b) Give reasons for non-participation at each stage	n/a
		(c) Consider use of a flow diagram	n/a
Descriptive data	14*	(a) Give characteristics of study participants (eg demographic, clinical, social) and information on exposures and potential confounders	Descriptive Statistics
		(b) Indicate number of participants with missing data for each variable of interest	Descriptive Statistics
Outcome data	15*	Report numbers of outcome events or summary measures	Results
Main results	16	(*a*) Give unadjusted estimates and, if applicable, confounder-adjusted estimates and their precision (eg, 95% confidence interval). Make clear which confounders were adjusted for and why they were included	Results
		(*b*) Report category boundaries when continuous variables were categorized	n/a
		(*c*) If relevant, consider translating estimates of relative risk into absolute risk for a meaningful time period	n/a
Other analyses	17	Report other analyses done—eg analyses of subgroups and interactions, and sensitivity analyses	Results
**Discussion**			
Key results	18	Summarise key results with reference to study objectives	Discussion
Limitations	19	Discuss limitations of the study, taking into account sources of potential bias or imprecision. Discuss both direction and magnitude of any potential bias	Strengths and Limitations
Interpretation	20	Give a cautious overall interpretation of results considering objectives, limitations, multiplicity of analyses, results from similar studies, and other relevant evidence	Discussion
Generalisability	21	Discuss the generalisability (external validity) of the study results	Strengths and Limitations
**Other information**			
Funding	22	Give the source of funding and the role of the funders for the present study and, if applicable, for the original study on which the present article is based	Funding

*Give information separately for exposed and unexposed groups.
